# Modulation of the Host Lipid Landscape to Promote RNA Virus Replication: The Picornavirus Encephalomyocarditis Virus Converges on the Pathway Used by Hepatitis C Virus

**DOI:** 10.1371/journal.ppat.1005185

**Published:** 2015-09-25

**Authors:** Cristina M. Dorobantu, Lucian Albulescu, Christian Harak, Qian Feng, Mirjam van Kampen, Jeroen R. P. M. Strating, Alexander E. Gorbalenya, Volker Lohmann, Hilde M. van der Schaar, Frank J. M. van Kuppeveld

**Affiliations:** 1 Department of Infectious Diseases & Immunology, Virology Division, Faculty of Veterinary Medicine, Utrecht University, Utrecht, The Netherlands; 2 Department of Infectious Diseases, Molecular Virology, University of Heidelberg, Heidelberg, Germany; 3 Department of Medical Microbiology, Leiden University Medical Center, Leiden, The Netherlands; 4 Faculty of Bioengineering and Bioinformatics, Lomonosov Moscow State University, Moscow, Russia; University of California, Irvine, UNITED STATES

## Abstract

Cardioviruses, including encephalomyocarditis virus (EMCV) and the human Saffold virus, are small non-enveloped viruses belonging to the *Picornaviridae*, a large family of positive-sense RNA [(+)RNA] viruses. All (+)RNA viruses remodel intracellular membranes into unique structures for viral genome replication. Accumulating evidence suggests that picornaviruses from different genera use different strategies to generate viral replication organelles (ROs). For instance, enteroviruses (e.g. poliovirus, coxsackievirus, rhinovirus) rely on the Golgi-localized phosphatidylinositol 4-kinase III beta (PI4KB), while cardioviruses replicate independently of the kinase. By which mechanisms cardioviruses develop their ROs is currently unknown. Here we show that cardioviruses manipulate another PI4K, namely the ER-localized phosphatidylinositol 4-kinase III alpha (PI4KA), to generate PI4P-enriched ROs. By siRNA-mediated knockdown and pharmacological inhibition, we demonstrate that PI4KA is an essential host factor for EMCV genome replication. We reveal that the EMCV nonstructural protein 3A interacts with and is responsible for PI4KA recruitment to viral ROs. The ensuing phosphatidylinositol 4-phosphate (PI4P) proved important for the recruitment of oxysterol-binding protein (OSBP), which delivers cholesterol to EMCV ROs in a PI4P-dependent manner. PI4P lipids and cholesterol are shown to be required for the global organization of the ROs and for viral genome replication. Consistently, inhibition of OSBP expression or function efficiently blocked EMCV RNA replication. In conclusion, we describe for the first time a cellular pathway involved in the biogenesis of cardiovirus ROs. Remarkably, the same pathway was reported to promote formation of the replication sites of hepatitis C virus, a member of the *Flaviviridae* family, but not other picornaviruses or flaviviruses. Thus, our results highlight the convergent recruitment by distantly related (+)RNA viruses of a host lipid-modifying pathway underlying formation of viral replication sites.

## Author Summary

All positive-sense RNA viruses [(+)RNA viruses] replicate their viral genomes in tight association with reorganized membranous structures. Viruses generate these unique structures, often termed “replication organelles” (ROs), by efficiently manipulating the host lipid metabolism. While the molecular mechanisms underlying RO formation by enteroviruses (e.g. poliovirus) of the family *Picornaviridae* have been extensively investigated, little is known about other members belonging to this large family. This study provides the first detailed insight into the RO biogenesis of encephalomyocarditis virus (EMCV), a picornavirus from the genus *Cardiovirus*. We reveal that EMCV hijacks the lipid kinase phosphatidylinositol-4 kinase IIIα (PI4KA) to generate viral ROs enriched in phosphatidylinositol 4-phosphate (PI4P). In EMCV-infected cells, PI4P lipids play an essential role in virus replication by recruiting another cellular protein, oxysterol-binding protein (OSBP), to the ROs. OSBP further impacts the lipid composition of the RO membranes, by mediating the exchange of PI4P with cholesterol. This membrane-modification mechanism of EMCV is remarkably similar to that of the distantly related flavivirus hepatitis C virus (HCV), while distinct from that of the closely related enteroviruses, which recruit OSBP via another PI4K, namely PI4K IIIβ (PI4KB). Thus, EMCV and HCV represent a striking case of functional convergence in (+)RNA virus evolution.

## Introduction

*Picornaviridae* is a large family of positive-sense RNA viruses [(+)RNA viruses] comprising many clinically relevant human and animal pathogens. Members of the genus *Enterovirus* include important human viruses like poliovirus (PV), the causative agents of poliomyelitis, coxsackieviruses (CV), causing meningitis and myocarditis, and rhinoviruses (RV), responsible for the common cold and exacerbations of asthma and chronic obstructive pulmonary disease. Perhaps the best-known non-human picornavirus is foot-and-mouth-disease virus (FMDV, genus *Aphtovirus*), which can cause devastating outbreaks in cattle leading to severe economic loss. Closely related to the *Apthovirus* genus is the genus *Cardiovirus*, composed of three species: *Theilovirus* (TV), *encephalomyocarditis virus* (EMCV) and the more recently discovered *Boone cardiovirus*. The species *Theilovirus* includes, among others, Theiler’s murine encephalomyocarditis virus (TMEV) and Saffold virus (SAFV), a human cardiovirus. While TMEV is known to cause enteric infections and sometimes more severe encephalitis or chronic infection of the central nervous system [1], as yet, SAFV has not been firmly associated with a clinical disease [2]. EMCV can infect a wide range of animals, of which rodents are considered the natural reservoir. Of all domesticated animals, pigs are most prone to EMCV infection, which can lead to fatal myocarditis [3], reproductive failure in sows or sudden death of piglets [4–6].

Like other (+)RNA viruses—such as hepatitis C virus (HCV), dengue virus (DENV), chikungunya virus (ChikV) and coronavirus (CoV)—picornaviruses replicate their genomic RNA on specialized, virus-modified intracellular membranes. These remodeled membranes termed replication organelles (ROs) arise from the concerted actions of both viral nonstructural proteins and co-opted host factors. Enteroviruses, for instance, hijack members of the secretory pathway for replication and formation of ROs [7,8]. Among the viral nonstructural proteins, 2B, 2C, 3A as well as their precursors 2BC and 3AB contain hydrophobic domains which confer them membrane-modifying properties [9–11]. Considerable interest has been given to the study of the small viral protein 3A, which is the key viral player involved in membrane rearrangements. 3A interacts with and recruits secretory pathway components GBF1 (Golgi-specific brefeldin A-resistance guanine nucleotide exchange factor 1) and PI4KB (phosphatidylinositol-4 kinase type III isoform β) to ROs [12–16]. Despite intensive investigation, the role of GBF1 in enterovirus replication is not yet elucidated (reviewed in [8]). Recruitment of PI4KB to ROs leads to a significant local increase of membranes in its enzymatic product PI4P [15]. This PI4P-rich environment serves to further recruit other essential viral and host factors to replication sites, such as the viral polymerase 3D^pol^, which is able to specifically bind PI4P *in vitro*. Recently, we and others revealed that PI4P plays a central role in enterovirus replication by recruiting the oxysterol-binding protein (OSBP) to ROs [17–19]. In uninfected cells, OSBP bridges the ER and Golgi membranes by binding to the ER integral membrane protein VAP-A and to PI4P and Arf1-GTP at the trans-Golgi [20]. Through its sterol-binding domain, OSBP shuttles cholesterol from ER to the Golgi and PI4P from the Golgi to the ER, thereby generating a lipid counterflow at ER-Golgi membrane contact sites (MCSs). In enterovirus infection, OSBP exchanges PI4P for cholesterol most likely at ER-RO MCSs [18]. The unique lipid and protein composition of enterovirus ROs determines their particular 3D architecture, which consists of a complex tubulo-vesicular network, as shown in cells infected with PV and coxsackievirus B3 (CVB3) [21,22].

The lipid transfer function of OSBP at membrane contact sites is not only vital for enteroviruses, but also for HCV [23]. HCV genome replication occurs in association with an ER-derived network of specialized membrane vesicles called the membranous web (MW). Like enterovirus ROs, the HCV MW is enriched in PI4P lipids and cholesterol [23–25]. In the case of HCV, PI4P are generated through recruitment and activation of the ER-localized enzyme PI4KA (phosphatidylinositol-4-phopshate kinase type III isoform α) by the viral protein NS5A [24,26].

Thus far, information regarding virus-host interactions that govern the formation of cardiovirus ROs remains scarce. In a report by *Zhang* et al, it was suggested that autophagy supports EMCV replication [27]. The study showed that EMCV infection triggered an accumulation of autophagosome-like vesicles in the cytoplasm and that EMCV 3A colocalized with the autophagy marker LC3. However, inhibition of autophagy exerted only minor effects on virus replication [27], which argues against a strong implication of the autophagy pathway in cardiovirus genome replication and/or formation of ROs. Evidence for a role of autophagy in virus replication also exists for enteroviruses and flaviviruses, but rather related to non-lytic virus release or modulation of host innate immune responses than viral genome replication [28–31].

Based on observations that cardioviruses do not require GBF1 or PI4KB for replication [32–34], it is generally believed that cardiovirus replication strategies are distinct from those of enteroviruses. Here, we set out to elucidate whether cardiovirus replication depends on another PI4K isoform. By siRNA-mediated knockdown, we identified PI4KA as a key player in the replication of EMCV. EMCV 3A interacts with and recruits PI4KA to ROs, which increases local PI4P synthesis, eventually leading to downstream recruitment of OSBP. We show that the cholesterol-PI4P shuttling activity of OSBP is important for the global distribution of the ROs and for virus genome replication. Our data reveal that, by exploiting the same cellular pathway, the cardiovirus replication strategy profoundly resembles that of the distantly related HCV and is dissimilar to those of other characterized picornaviruses and flaviviruses in this critical aspect. Thus, the similarity between EMCV and HCV is a striking case of functional convergence in virus-host interactions, indicating that diverse RNA viruses might have a limited choice of pathways in the remodeling of host membrane network for virus replication.

## Results

### Cardiovirus replication requires PI4KA

**Fig 1:**
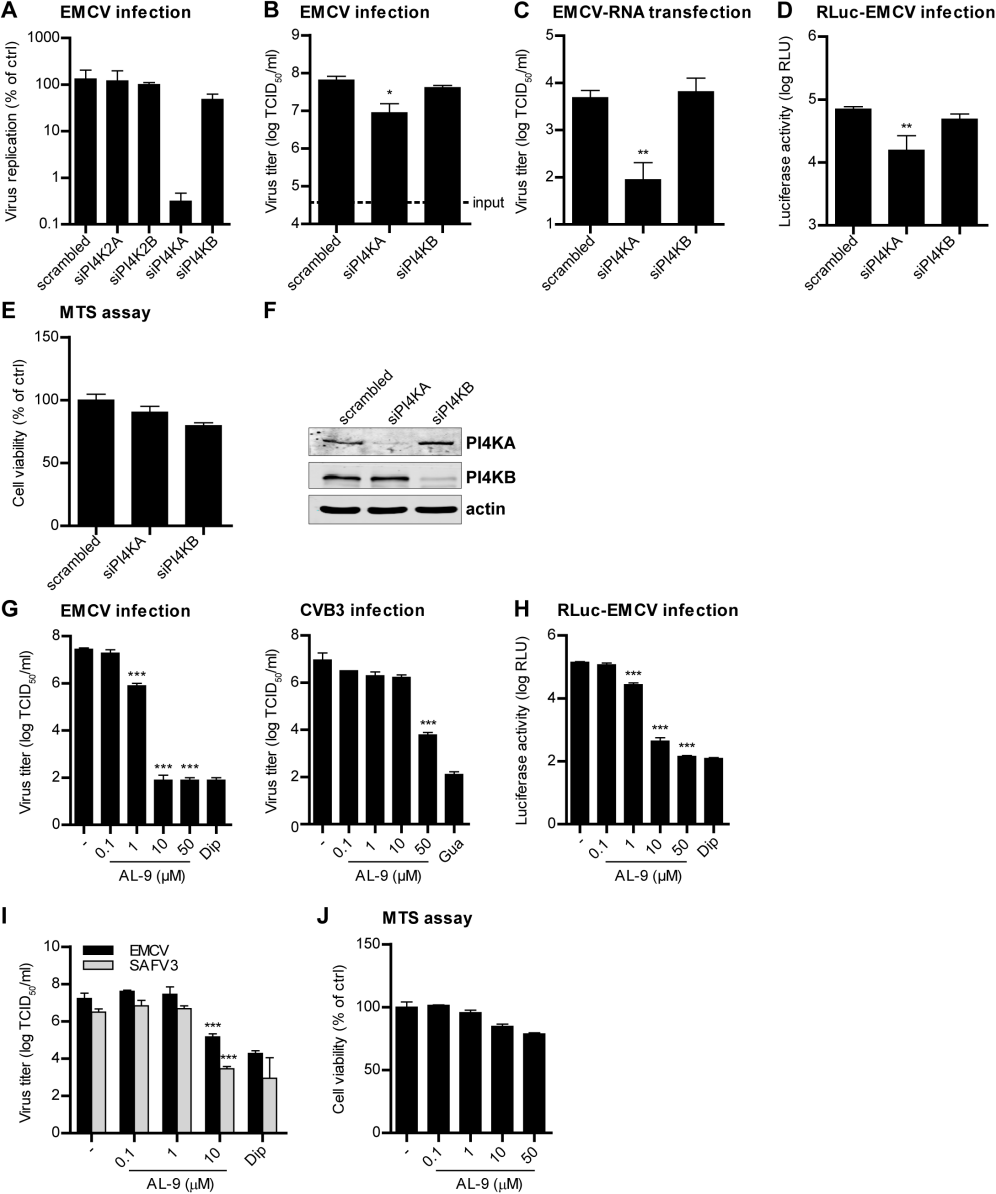
PI4KA is essential for cardiovirus replication. (A-D) Effects of PI4KA knockdown on EMCV infection. HeLa R19 cells were reverse transfected with siRNA against PI4K2A, PI4K2B, PI4KA, PI4KB or scrambled siRNA as a control (A) or siRNA against PI4KA, PI4KB or scrambled siRNA (B-D). At 48 h post transfection (p.t.), cells were infected with EMCV (A-C) or RLuc-EMCV (D) at an MOI of 0.1 or transfected with full-length infectious EMCV *in vitro*-transcribed RNA (C). After 8 h, cells were freeze-thawed to release intracellular virus particles and the total virus titers were determined by endpoint titration (A-C). Alternatively, cells were lysed and *Renilla* luciferase activity was determined as a measure of viral RNA replication (D). In parallel, a cell viability assay was performed to evaluate the cytotoxicity of the siRNA treatment (E). (F) Western blot analysis showing efficient knockdown of PI4KA and PI4KB. Actin was used as loading control. (G-I) AL-9 treatment inhibits cardiovirus replication. HeLa R19 cells were infected with virus at an MOI of 0.1 (G and H) or 1 (I), followed by AL-9 treatment for 8 h, after which cells were lysed and virus replication was measured by endpoint titration (G and I) or by determining the *Renilla* luciferase activity (H). Cytotoxicity of AL-9 was determined in a cell viability assay run in parallel (J). Bars represent mean values of triplicates ± standard error of the means (SEM). Means were statistically compared using unpaired *t* tests. *P < 0.05, **P<0.01; ***P<0.001.

Unlike enteroviruses, cardioviruses do not require PI4KB for replication [34]. To investigate whether other PI4Ks might be involved in cardiovirus replication, we depleted each of the four distinct cellular PI4Ks by siRNA-mediated gene knockdown using a set of siRNA sequences (Ambion) which we previously tested for efficiency and toxicity [35], and monitored the subsequent effects on replication of EMCV. We observed inhibitory effects on EMCV replication when silencing PI4KA, but not upon silencing of the other PI4Ks (Fig 1A). To confirm the importance of PI4KA for EMCV replication, we performed another series of knockdown experiments using another set of siRNA sequences (Qiagen). Depletion of PI4KA, but not PI4KB, significantly reduced EMCV infection, measured by end-point titration of progeny virus production (Fig 1B).

We next wondered which step in the virus life cycle is dependent on PI4KA. To omit the step of virus attachment and cell entry, EMCV RNA was *in vitro* transcribed and subsequently transfected in cells depleted of PI4KA by siRNAs. Virus replication was strongly inhibited upon PI4KA silencing, as measured by end-point titration of progeny virions (Fig 1C). This indicated that PI4KA is involved in a post-entry step in the virus life cycle. To elucidate whether EMCV requires PI4KA for viral genome amplification, we infected cells with a *Renilla* luciferase-encoding EMCV (RLuc-EMCV) and quantified the luciferase activity as a measure of viral RNA replication. EMCV RNA replication was severely impaired in cells lacking PI4KA, but not PI4KB (Fig 1D). We excluded that inhibition of EMCV replication by PI4KA silencing was due to cytotoxic effects by a cell viability assay (Fig 1E) and verified the knockdown efficiency by western blot analysis (Fig 1F). Altogether, these results showed that PI4KA plays a key role in EMCV genome RNA replication.

Next, we investigated whether EMCV required the enzymatic activity of PI4KA using AL-9, a PI4K inhibitor that also blocks PI4KB, but at 5-fold higher concentration [36]. Cells were infected with EMCV or RLuc-EMCV at MOI 0.1 and treated with increasing concentrations of AL-9 for 8 h. Coxsackievirus B3 (CVB3), as well as all other enteroviruses, has been previously shown to hijack the Golgi-localized PI4KB for replication [15,34] and was included as a control. As measured by end-point titration and analysis of the luciferase activity (Fig 1G and 1H), EMCV replication was efficiently inhibited by AL-9 in a dose-dependent manner with complete inhibition detected at 10 μM, while CVB3 replication was hampered only at 50 μM (Fig 1G), which is in line with the 5-fold preference of AL-9 for PI4KA over PI4KB. Dipyridamole, a well-established inhibitor of EMCV RNA replication, was included here as positive control. Importantly, AL-9 inhibited EMCV replication also when infection was performed at high MOI (S1A Fig, MOI 10). To corroborate that PI4KA activity is required for the step of viral genome replication, we performed a time-of-addition experiment in which AL-9 was added to the cells at different time points after infection with RLuc-EMCV. Similar to dipyridamole, AL-9 strongly inhibited replication when added up to 3 h after infection (S1B Fig), indicating that not entry but rather a step during genome replication was blocked by AL-9.

Next, we tested whether other members of the cardiovirus genus also depended on PI4KA for replication. Similar to EMCV, replication of the human cardiovirus Saffold virus 3 (SAFV3) (species *Theilovirus*) was also sensitive to AL-9 treatment (Fig 1I). The cell viability assay demonstrated that AL-9 treatment only exerted slight cytotoxic effects at the highest concentration tested (Fig 1J). These results indicated that different cardiovirus species required the enzymatic activity of PI4KA for genome replication.

### PI4KA localizes to EMCV replication sites

**Fig 2:**
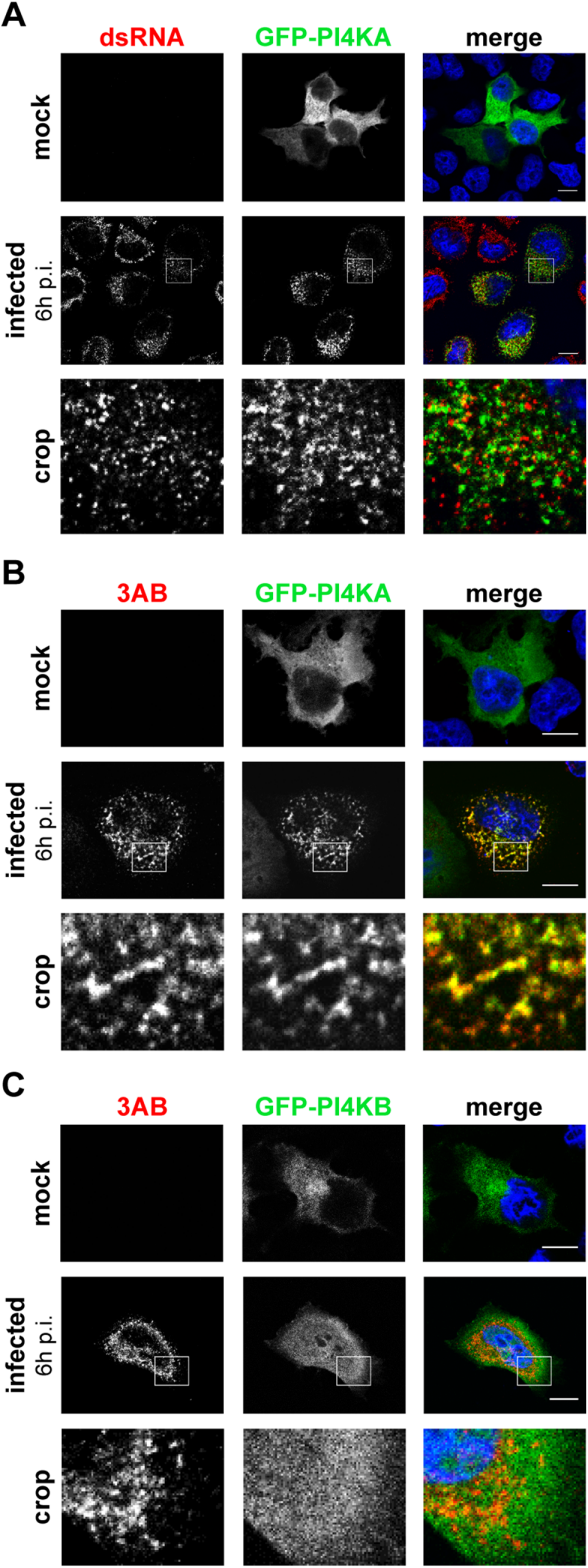
PI4KA is recruited to EMCV replication sites. HeLa R19 cells were transfected with plasmids encoding GFP-PI4KA (A and B) or GFP-PI4KB (C). The next day, cells were mock-infected or infected with EMCV at an MOI of 250. At 6 h post infection (p.i.), cells were fixed and stained with antibodies against dsRNA as a marker of infection (A) or 3AB as a RO marker (B and C). Nuclei were stained with DAPI (blue). The crop panels at the bottom depict enlargements of boxed areas. Scale bars represent 10 μm.

Soon after infection, the cytoplasm of EMCV-infected cells accumulates an impressive amount of vesicular membranous structures [37,38]. As yet, there is little information available regarding which viral proteins and host factors are associated with these new virus-induced organelles [27,39]. We set out to investigate whether PI4KA was present at EMCV ROs. Despite repeated efforts, we were unable to detect the endogenous kinase by immunofluorescence staining in any of the cell lines tested. As an alternative, we chose to analyze possible changes in the subcellular distribution of ectopically expressed PI4KA upon EMCV infection. In mock-infected cells (Fig 2A, upper panel), GFP-PI4KA was distributed diffusely throughout the entire cytoplasm, as previously reported by others [40,41]. In infected cells visualized by dsRNA staining, we instead observed a clear difference in the localization of the kinase, which was redistributed to discrete cytoplasmic punctae in a perinuclear region (Fig 2A, lower panel).

We next aimed to elucidate whether these PI4KA punctae coincided with the viral ROs. The small picornaviral protein 3A and its precursor 3AB are membrane-associated and play key roles in viral RNA replication and recruitment of essential host factors [15,42–45]. Hence, we considered 3AB as a suitable marker for EMCV ROs and compared the staining of PI4KA to that of 3AB in infected cells. We observed a striking overlap of GFP-PI4KA with 3AB-positive structures (Fig 2B, lower panels) and could confirm this phenotype when analyzing the localization of ectopically expressed PI4KA bearing an HA-tag (S2 Fig). By contrast, the signal for GFP-PI4KB, which was mainly localized at the Golgi in non-infected cells (Fig 2C, top panel), failed to overlap with 3AB (Fig 2C, lower panels). Interestingly, although in close proximity to dsRNA signals (Fig 2A, lower panel), PI4KA did not clearly overlap with dsRNA (Fig 2A, insets). Taken together, these data demonstrated that PI4KA is selectively recruited to EMCV ROs.

**Fig 3:**
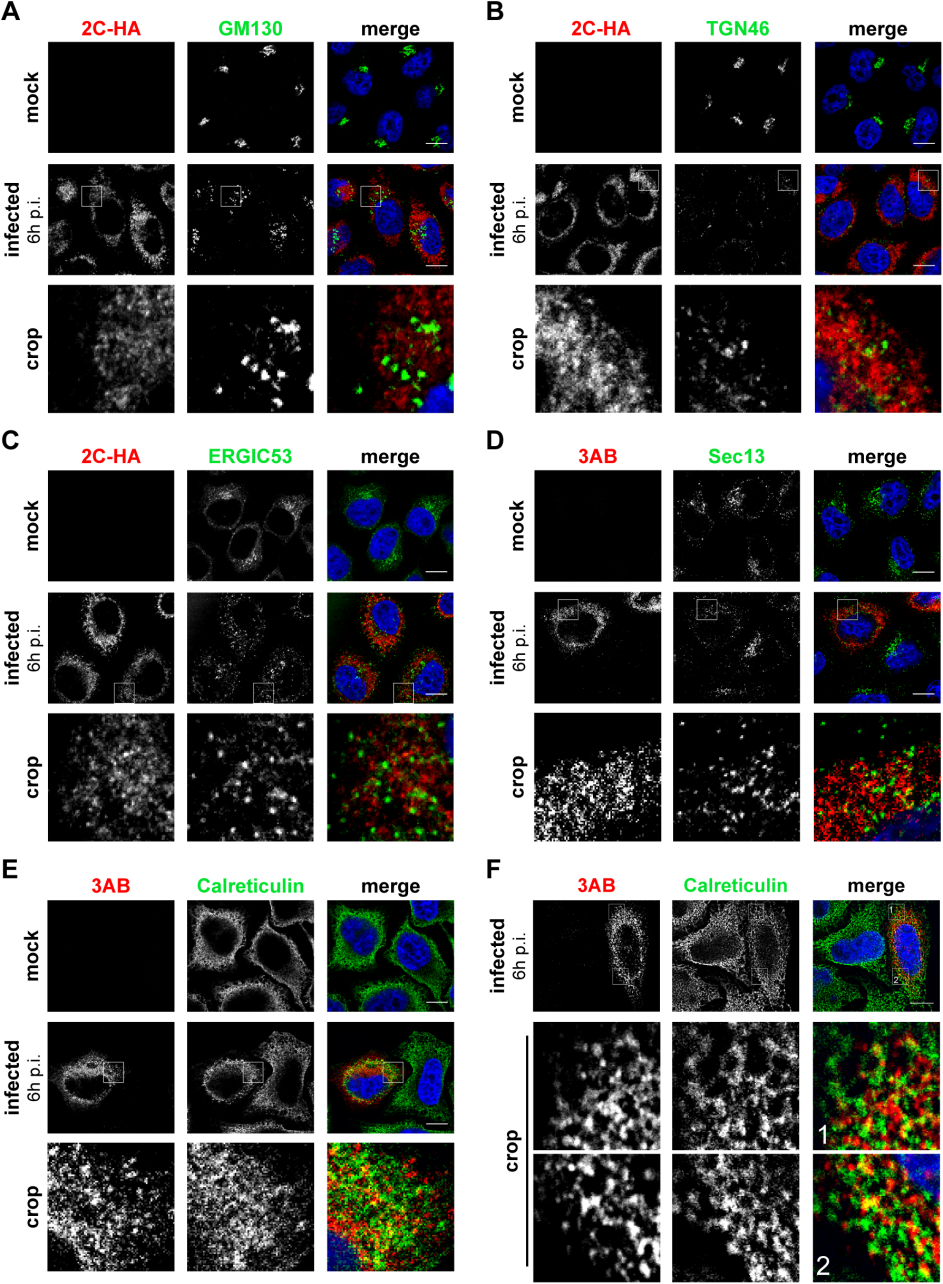
Localization of early secretory membranes in EMCV-infected cells. HeLa R19 cells were mock-infected or infected with EMCV-2C-HA (A-C) or EMCV (D-F) at MOI 10. 6 h later, cells were fixed and co-stained with antibodies against viral 3AB or HA as RO markers and antibodies against endogenous GM130 (*cis*-Golgi marker; A), TGN46 (*trans*-Golgi marker, B), ERGIC53 (ERGIC marker, C), Sec13 (ER exit site marker, D) or calreticulin (ER marker, E and F). Nuclei were stained with DAPI (blue). The crop panels at the bottom depict enlargements of boxed areas. Scale bars represent 10 μm.

Interestingly, we noticed a loss of the typical Golgi localization of PI4KB in EMCV-infected cells (Fig 2C, lower panel), suggesting that Golgi integrity might be affected upon EMCV infection. Prompted by this and our finding that EMCV utilizes the ER-localized PI4KA for replication, we set out to elucidate whether other ER or Golgi components are present at EMCV ROs. In order to be able to use more antibody combinations in immunofluorescence, we constructed a recombinant EMCV bearing an HA-tag in the nonstructural protein 2C. The tag was introduced after the second amino acid, leaving the 2B-2C cleavage site intact (S3A Fig), and did not impair virus replication (S3B Fig). First, we checked whether 2C-HA and 3AB are present on the same membranes by immunofluorescence microscopy. Indeed, 2C and 3AB signals greatly overlapped (S3C Fig), supporting the idea that these proteins occupy the same membranes of the ROs. Using this tagged EMCV, we noticed that the Golgi structure was indeed altered in infected cells, from 4 h p.i. onwards, as revealed by the dispersed pattern of both *cis*- and *trans*-Golgi markers GM130 (Fig 3A) and TGN46, respectively (Fig 3B). However, neither TGN46 nor GM130 were present at 2C-HA-positive structures, suggesting that EMCV ROs are not Golgi-derived. ERGIC53, a marker of the ER-Golgi intermediate compartment also appeared scattered throughout the cytoplasm in infected cells, but without overlapping 2C-HA (Fig 3C). We next compared the localization of 3AB with Sec13 (COPII-coatomer complex component), an ER exit site (ERES) marker, and the ER marker calreticulin. While in non-infected cells, Sec13 displayed mainly a typical perinuclear localization, in EMCV-infected cells it appeared dispersed, but without significantly colocalizing with 3AB (Fig 3D, Mander’s colocalization coefficient M2 = 0.14 ± 0.01, fraction of Sec13 overlapping 3AB). We observed a greater degree of overlap between 3AB and calreticulin (Fig 3E, M2 = 0.4 ± 0.02, fraction of calreticulin overlapping 3AB). Images acquired with higher magnification revealed that most of 3AB was in close contact with ER tubules (Fig 3F). Taken together, these data suggested that EMCV possibly replicates on ER-derived membranes.

### EMCV 3A interacts with PI4KA

**Fig 4:**
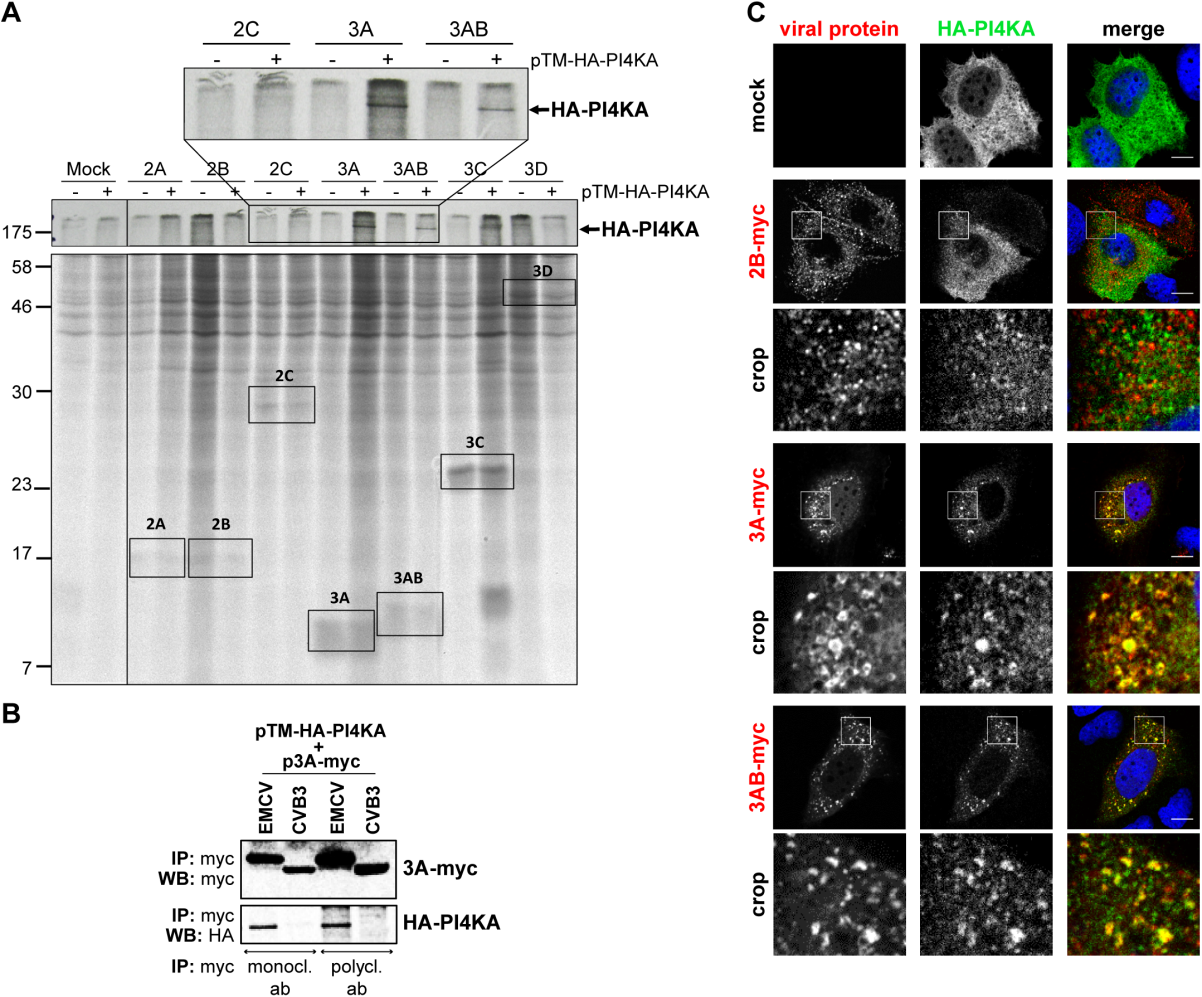
EMCV 3A interacts with PI4KA. (A) Only EMCV 3A and 3AB can interact with PI4KA. Huh7-Lunet/T7 cells were transfected with empty vector or plasmids encoding myc-tagged EMCV nonstructural proteins and co-transfected with pTM-HA-PI4KA where indicated. 7 h later, cells were radiolabeled for 16 h with [^35^S] methionine/cysteine-containing medium, lysed and subjected to immunoprecipitation using anti-myc antibodies. Samples were analyzed by SDS-PAGE and visualized by autoradiography. An enlargement of the boxed area at the top shows co-precipitation of HA-PI4KA with 3A- and 3AB-myc, but not with 2C-myc. (B) Huh7-Lunet/T7 cells were transfected with plasmids encoding HA-PI4KA and either myc-tagged EMCV 3A or CVB3 3A proteins. One day later, cells were lysed and subjected to immunoprecipitation using two different anti-myc antibodies. Captured complexes were separated by SDS-PAGE and subjected to western blot analysis using specific antibodies against the myc- or HA-tags. (C) Specific recruitment of PI4KA to 3A-positive membranes. Huh7-Lunet/T7 cells were cotransfected with the HA-PI4KA expression construct and either empty vector or plasmids encoding myc-tagged EMCV 2B, 3A or 3AB. The next day, cells were fixed and co-stained with antibodies against myc and HA to detect the overexpressed proteins. The crop panels at the bottom depict an enlargement of the boxed areas. Scale bars represent 10 μm.

Based on the extensive overlap between PI4KA and 3AB and the drastic change in PI4KA pattern in infected cells, we hypothesized that PI4KA might be recruited to replication sites by interacting (directly or indirectly) with one or more of the viral nonstructural proteins. To investigate this, we used the stable cell line Huh7-Lunet/T7 that allows ectopic protein expression under the control of a T7 promoter and has been previously optimized and validated as a reliable and reproducible cellular system to study PI4KA-protein interactions by radioactive Co-IP assays [40,46]. Myc-tagged EMCV nonstructural proteins 2A, 2B, 2C, 3A, 3AB, 3C and 3D were individually co-expressed together with HA-PI4KA in Huh7-Lunet/T7 cells, radioactively labeled, and affinity purified from cell lysates using anti-myc specific antibodies. Autoradiography analysis showed that HA-PI4KA was specifically co-purified by 3A and 3AB, but not by the other viral proteins (Fig 4A). To confirm this interaction by co-immunoprecipitation (co-IP) coupled with western blot analysis, myc-tagged EMCV 3A was co-expressed with HA-PI4KA and subjected to affinity purification using either monoclonal or polyclonal anti-myc antibodies. As shown in Fig 4B, HA-PI4KA only interacted with EMCV 3A, but not with CVB3 3A, which interacts with PI4KB [15,16,45] and was included here as a negative control. These data implied that EMCV nonstructural protein 3A is responsible for PI4KA recruitment to ROs. Interestingly, a diffuse band just below 17 KDa appears to co-purify with EMCV 3C when HA-PI4KA is co-expressed (Fig 4A, indicated by *). We reasoned this could be indicative of a temporal regulation of the PI4KA activity during infection via 3C-dependent degradation. To explore this possibility, we performed western blot analysis of endogenous PI4KA during the time course of infection, but did not detect any bands indicative of degradation, neither in Huh7-Lunet/T7 or HeLa R19 cells (S4 Fig). To test if 3A alone can recruit PI4KA, we examined by immunofluorescence the subcellular localization of HA-PI4KA when co-expressed with 3A, 3AB or 2B, which we considered as a negative control. When expressed alone, HA-PI4KA localized throughout the cell in a diffuse pattern (Fig 4C, top panel), as previously described [40]. EMCV 3A- and 3AB-myc were both localized throughout the cytoplasm and at discrete punctate structures, of which a subset was also positive for PI4KA (Fig 4C). 2B-myc was also distributed in punctae throughout the cytoplasm, but failed to recruit PI4KA (Fig 4C). Collectively, these results indicated that EMCV 3A is the viral protein responsible for engaging PI4KA in replication.

### EMCV alters PI4P homeostasis

**Fig 5:**
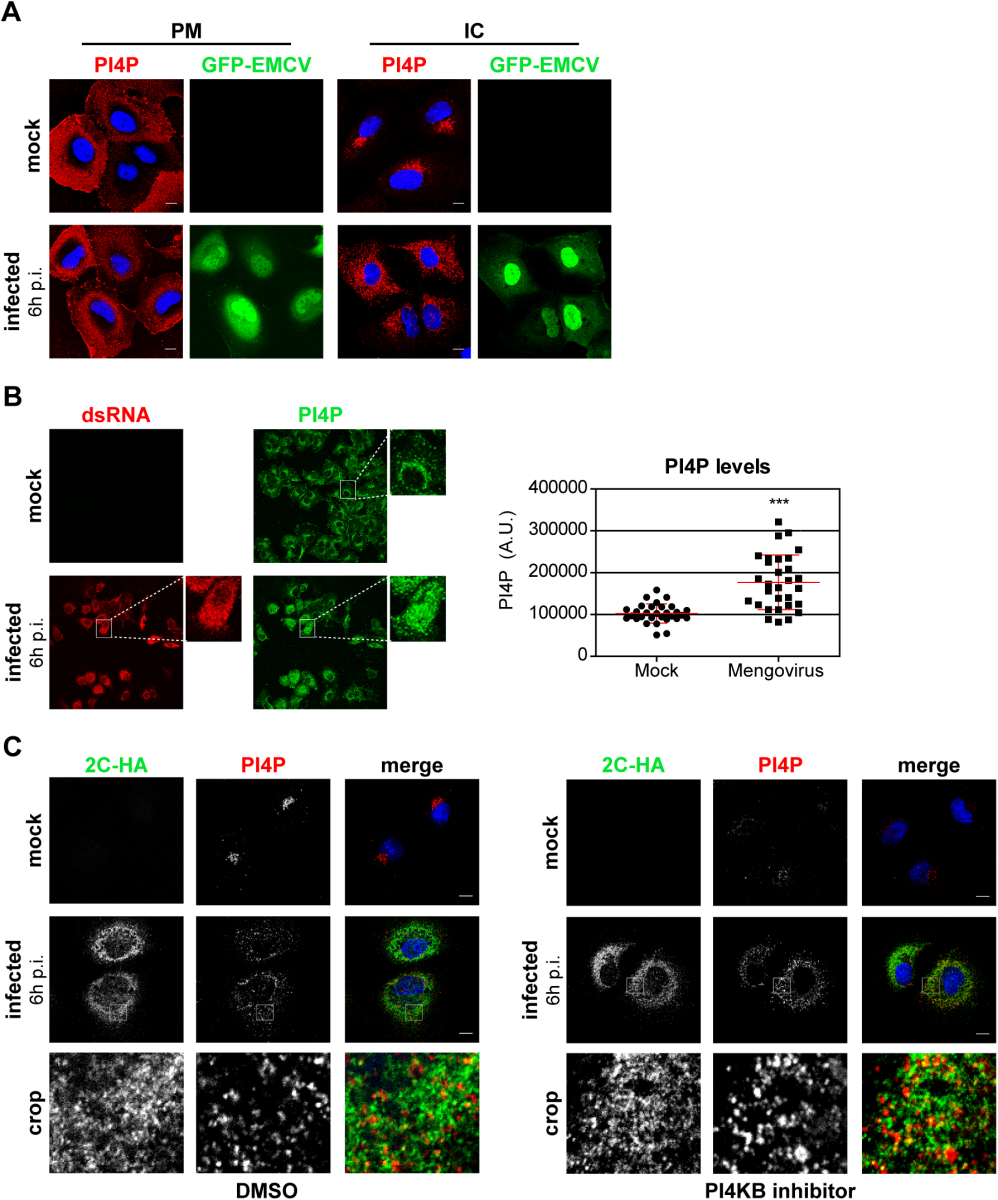
PI4P homeostasis is affected upon EMCV infection. (A) EMCV alters the distribution of intracellular PI4P lipids. Huh7-Lunet/T7 cells were mock-infected or infected with GFP-EMCV at an MOI of 10. At 6 h p.i., cells were fixed and stained with antibodies against PI4P using specific protocols for detection of plasma membrane and intracellular PI4P pools, as described previously [50]. Nuclei were stained with DAPI (blue). (B) Quantification of PI4P levels by immunofluorescence analysis. Huh7-Lunet/T7 cells were mock-infected or infected with EMCV at an MOI of 10. At 6 h p.i., cells were fixed and stained for intracellular PI4P and viral dsRNA. The intensities of fluorescent PI4P signals from whole-cell z-stacks were quantified using ImageJ. Shown are mean values ± SEM of 30 cells per condition. Means of mock and infected cells were statistically analyzed using the Mann–Whitney test. ***P<0.001. (C) EMCV ROs contain PI4P. Huh7-Lunet/T7 cells were mock-infected or infected with EMCV-2C-HA at an MOI of 10. At 5.5 h p.i., cells were treated with DMSO or 1 μM BF738735 (PI4KB inhibitor) for 30 min, then fixed and stained for intracellular PI4P and HA using specific antibodies. Nuclei were stained with DAPI (blue). The crop panels at the bottom depict an enlargement of the boxed areas. Scale bars represent 10 μm.

While PI4KB produces PI4P at Golgi membranes, PI4KA is responsible for the synthesis of the PI4P pool at the plasma membrane, where it dynamically localizes [41,47–49]. Our finding that PI4KA activity was critical for EMCV RNA replication prompted us to investigate whether PI4P metabolism is altered during virus replication. Given that EMCV replicates on intracellular membranes, we monitored potential changes in the subcellular distribution of both plasma membrane (PM) and intracellular (IC) pools of PI4P in Huh7Lunet/T7 cells following EMCV infection. The two pools of PI4P can be selectively visualized using two different immunocytochemistry protocols previously established by Hammond et al [50]. While the plasma membrane pool of PI4P appeared unaffected in EMCV-infected cells (Fig 5A), the intracellular PI4P distribution changed from a perinuclear, Golgi-like pattern in mock-infected cells to dispersed throughout the cytoplasm in EMCV-infected cells (Fig 5A). We observed similar PI4P phenotypes in HeLa cells (S5 Fig), indicating that the observed effects were not cell line-specific. Notably, quantitative analysis of the fluorescent PI4P signals revealed a marked increase in the level of intracellular PI4P in infected cells (Fig 5B).

To rule out a possible involvement of PI4KB in establishing the elevated PI4P levels observed in EMCV infected cells, we treated cells with the PI4KB inhibitor BF738735 (Compound 1) [34]. For simultaneous detection of PI4P and viral ROs by immunofluorescence, we infected cells with EMCV-2C-HA. Short treatment with BF738735 severely depleted the Golgi PI4P pool in non-infected cells (Fig 5C), thus reflecting an effective inhibition of PI4KB activity. However, the PI4P phenotype remained unaltered in infected cells (Fig 5C), demonstrating that the EMCV-induced accumulation of intracellular PI4P was not mediated by PI4KB. Most PI4P localized in the vicinity of 2C-HA, with at least a small subset of PI4P overlapping with 2C-HA (Fig 5C). These data together with the finding that EMCV requires PI4KA activity suggested that PI4KA-derived PI4P lipids play a central role in EMCV genome replication.

### OSBP supports EMCV genome replication downstream of PI4KA

**Fig 6:**
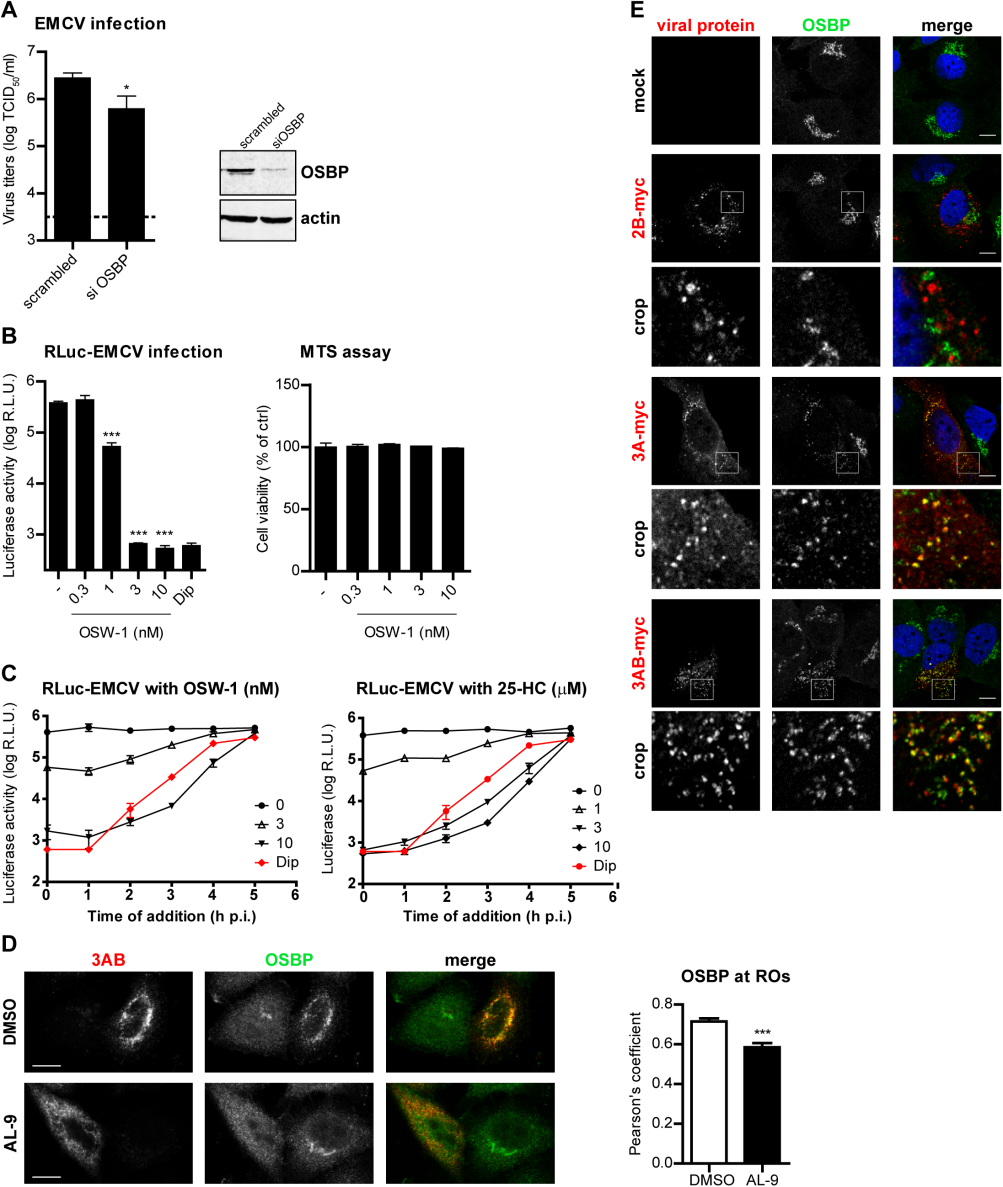
OSBP is a PI4KA effector important for EMCV replication. (A) OSBP knockdown reduces EMCV replication. HeLa R19 cells were reverse transfected with siRNA against OSBP or scrambled siRNA as a control. At 48 h p.t., cells were infected with EMCV at MOI 0.1. After 8 h, cells were freeze-thawed to release intracellular virus particles and total virus titers were determined by endpoint titration. OSBP knockdown efficiency was verified by western blot analysis. Actin was used as loading control. (B) Antiviral effects of the OSBP inhibitor OSW-1 against EMCV. HeLa R19 cells were infected with RLuc-EMCV at MOI 0.1, followed by treatment with OSW-1 at the indicated concentrations. *Renilla* luciferase levels at 8 h p.i were determined as a measure of virus genome replication. Cell viability was measured in parallel. (C) HeLa R19 cells were infected with RLuc-EMCV at an MOI of 1 and treated at the indicated time points after infection with DMSO, OSW-1 or 25-HC at the specified concentrations. At 7 h p.i., cells were lysed and *Renilla* luciferase activity was measured. (D) OSBP localization at EMCV ROs is PI4P-dependent. HeLa R19 cells were infected with EMCV at MOI 10. At 5.5 h p.i., cells were treated for 30 min with DMSO or 10 μM AL-9 to inhibit PI4KA activity, then fixed and subjected to immunofluorescence analysis using antibodies against OSBP and viral 3AB. Colocalization of OSBP with 3AB was determined by calculating the Pearson’s correlation coefficients for at least 15 cells for each condition. (E) Specific recruitment of OSBP to 3A-positive membranes. Huh7-Lunet/T7 cells were transfected with empty vector or plasmids encoding myc-tagged EMCV 2B, 3A or 3AB. One day later, cells were fixed and co-stained with antibodies against myc and endogenous OSBP. The crop panels at the bottom depict enlargements of boxed areas. Scale bars represent 10 μm. Shown are mean values ± SEM. Means were statistically compared using either unpaired *t* tests (A and B) or the Mann–Whitney test (D). *P < 0.05, ***P<0.001.

Various cellular proteins carrying a PI4P-binding domain called the pleckstrin-homology domain (PH), such as the ceramide-transfer protein (CERT), four-phosphate-adaptor protein 1 (FAPP1), or the oxysterol-binding protein (OSBP) can sense and specifically bind PI4P lipids [47,51–53]. Recently, we and others showed that enteroviruses generate PI4P-enriched membranes to recruit OSBP, which in turn exchanges PI4P for cholesterol at ROs [17,18,54]. Moreover, we showed that EMCV is sensitive to itraconazole, which we identified to be an OSBP inhibitor [17], and that cholesterol shuttling is important for EMCV replication [54]. We therefore reasoned that in EMCV-infected cells one purpose of PI4P lipids might be to recruit OSBP to replication membranes to support viral RNA replication. To test if OSBP is required for EMCV replication, we efficiently reduced OSBP expression in HeLa cells by siRNA gene silencing (Fig 6A) and evaluated the subsequent effects on EMCV replication by end-point titration analysis. Replication of EMCV was significantly reduced in cells in which OSBP was depleted compared to control-treated cells (Fig 6A), indicating that OSBP is required for efficient replication. We further used OSW-1, an OSBP ligand that interferes with normal OSBP functioning [55], to pharmacologically inhibit OSBP and analyze whether its lipid transfer function is linked to EMCV infection. Using luciferase-encoding EMCV, we observed a complete inhibition of genome RNA replication after 7 h of treatment with OSW-1 at nanomolar concentrations, with no cytotoxicity present (Fig 6B). A similar inhibition by OSW-1 was observed when infection was performed at high MOI (S6 Fig, MOI 10). Furthermore, by performing OSW-1 time-of-addition experiments, we excluded the possibility that OSBP was involved in early steps in the virus life cycle (Fig 6C). Similar results were obtained when using 25-hydroxycholesterol (25-HC, Fig 6C), another established OSBP ligand [20,56].

Next, we wondered whether endogenous OSBP was present at EMCV ROs and if so, whether this localization was dependent on the PI4P pool generated by PI4KA. To this end, cells were infected with EMCV for 5.5 h and then treated with DMSO or AL-9 for 30 min to acutely deplete PI4P, prior to immunofluorescence analysis. While in non-infected cells OSBP localized throughout the cytoplasm and at the Golgi, OSBP was mainly found at ROs in infected cells, where it largely colocalized with 3AB (Fig 6D, Pearson’s correlation coefficient = 0.71). Since other Golgi proteins were not present at the ROs (Fig 3A and 3B), these results suggested that OSBP is specifically recruited by EMCV. Following inhibition of PI4KA by short treatment with AL-9, we observed a strong and significant reduction of OSBP and 3AB colocalization (Fig 6D, Pearson’s coefficient = 0.58). Importantly, the subcellular localization of OSBP in non-infected cells was not affected by AL-9 treatment (Fig 6D), demonstrating that the presence of OSBP at EMCV replication structures is conditioned by PI4KA-produced PI4P.

Given the colocalization of OSBP with 3AB, we sought to verify whether EMCV 3A was responsible for OSBP recruitment. To this end, myc-tagged EMCV 3A-, 3AB- or 2B-myc were ectopically expressed in Huh7-Lunet/T7 cells and recruitment of endogenous OSBP was analyzed by immunofluorescence analysis. In cells expressing 3A and 3AB, OSBP was redistributed in punctate structures throughout the cytoplasm, with some of these punctae colocalizing with 3A/3AB (Fig 6E). By contrast, OSBP remained localized at the Golgi and did not localize at 2C-positive punctae (Fig 6E). These data indicated that during EMCV infection, OSBP is recruited to ROs via 3A.

### OSBP transfers cholesterol to EMCV ROs in a PI4P-dependent manner

**Fig 7:**
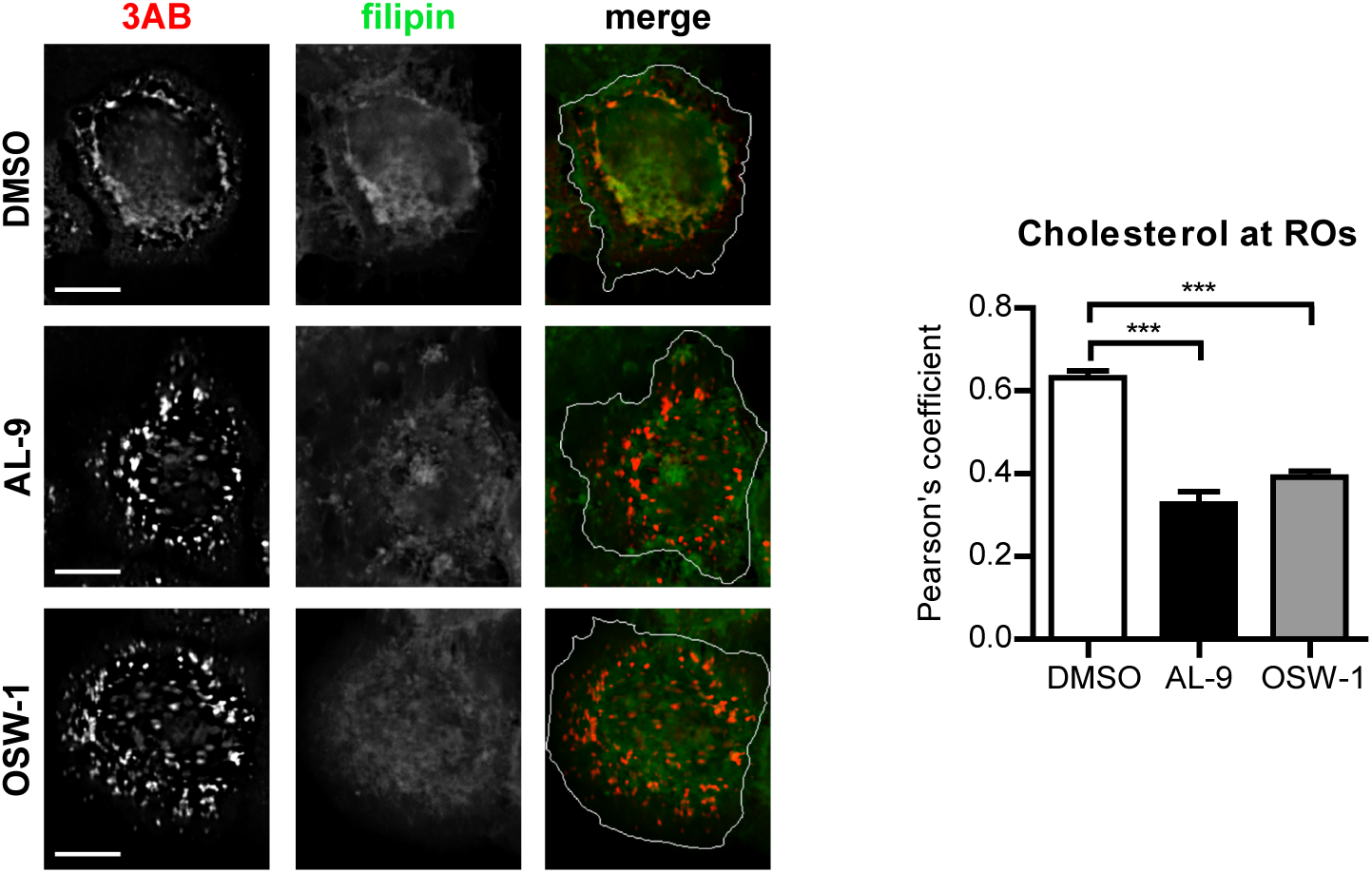
Cholesterol delivery to EMCV ROs depends on PI4KA and OSBP activities. (A) HeLa R19 cells were infected with EMCV at MOI 10. At 4 h p.i., cells were treated with DMSO, 10 μM AL-9 or 3nM OSW-1. After 2 h, cells were fixed, followed by staining with antibodies against 3AB or filipin for cholesterol detection. The merged panels also depict the outline of the cell (white line). Scale bars represent 10 μm. Pearson’s correlation coefficients of colocalization between filipin and 3AB were calculated for at least 15 cells for each condition. Shown are mean values ± SEM. Means were statistically compared using the Mann–Whitney test. ***P<0.001.

To test whether OSBP is involved in transferring cholesterol to ROs in a PI4P-dependent manner, cells were infected with EMCV for 4 h, treated with AL-9 or OSW-1 for 2 h to block PI4KA activity or OSBP function respectively, and subjected to immunofluorescence analysis. In non-infected cells, cholesterol mainly localizes at endosomes in the perinuclear area and at the plasma membrane, as visualized by filipin staining [54]. In infected cells treated with DMSO, we detected cholesterol primarily colocalizing with 3AB-positive structures (Pearson’s coefficient = 0.62), while in drug-treated cells this colocalization was markedly reduced (Fig 7, Pearson’s coefficient = 0.32 for AL-9 and 0.39 for OSW-1). This result confirmed that EMCV ROs acquire cholesterol through the actions of both PI4KA and OSBP.

## Discussion

(+)RNA viruses display immense genetic diversity, yet they all rely on remodeled membranes for viral genome replication. A diverse array of cellular organelles can be remodeled into viral replication structures. For instance, picornaviruses from the genera *Enterovirus* and *Kobuvirus* are thought to replicate at modified Golgi membranes [15,45,57], while the flavivirus HCV replicates on a membranous web originated from the ER [58]. To do so, viruses rewire host pathways involved in lipid synthesis and transport to generate replication membranes with unique lipid signatures [59–62]. How picornaviruses from the genus *Cardiovirus* build their ROs is currently unknown. Here, we identified PI4KA and OSBP as essential host factors for genome replication of the cardiovirus EMCV. Our data suggest that EMCV ROs may be derived from the ER and that PI4KA was recruited to ROs by interacting with the viral protein 3A(B). PI4KA recruitment led to a significant increase of intracellular PI4P levels in infected cells, which proved important for the downstream recruitment of OSBP. Finally, data are presented suggesting that the OSBP-mediated exchange of PI4P and cholesterol at RO-MCSs is critical for EMCV genome replication and the global organization of ROs.

Membrane alterations in the cytoplasm of cardiovirus-infected cells were already observed decades ago by electron microscopy [37,38,63]. As also described for enteroviruses, cardiovirus-induced membranes consist of perinuclear clusters of heterogeneous single- and double-membrane vesicles (DMVs). While recent studies greatly contributed to our understanding of the origin and biogenesis of enterovirus ROs [18,19,21,22,64,65], for cardioviruses these details have remained scarce. In a report by *Zhang* et al it was proposed that EMCV subverts the autophagy pathway to promote virus replication and RO formation [27]. The authors observed induction of autophagy and accumulation of cytoplasmic double-membrane vesicles (DMVs), a hallmark of autophagosomes, upon EMCV infection. However, inhibition of autophagy had stronger effects on extracellular than intracellular virus yields, which pointed towards a role of autophagy in virus release. Indeed, recent studies using enteroviruses and flaviviruses support the hypothesis that autophagy-derived membranes rather serve as means of non-lytic virus release and spread than as a membrane source for the viral ROs [28–31].

As opposed to enteroviruses, members of the *Cardiovirus* genus are insensitive to GBF1 depletion by siRNA [32] or treatment with BFA [33,39,66], a compound that targets GBF1 and subsequently blocks activation of Arf1, a key regulator of membrane trafficking in the secretory pathway. Furthermore, cardiovirus replication does not require the Golgi-localized PI4KB, which is essential for enterovirus replication [34]. Collectively, these data suggested that cardioviruses do not rely on Golgi components for replication. In line with these previous findings, we here present data suggesting that EMCV may derive its ROs from ER membranes. Confocal microscopy analysis revealed that EMCV nonstructural proteins partially overlapped with the ER marker calreticulin, but not with markers of ERES, ERGIC, *cis*- or *trans*-Golgi network, which appeared dispersed in infected cells, even at the earliest stages of infection. Furthermore, PI4KA, which normally resides at the ER network, was redistributed in EMCV-infected cells to discrete cytoplasmic structures that also contained the viral protein 3AB. Interestingly, the majority of PI4KA-positive punctae were detected in close proximity to viral dsRNA, but did not completely overlap with dsRNA, suggesting a spatial segregation of dsRNA from the viral replication membranes, previously also shown for coronaviruses [67,68].

### PI4P and cholesterol are key lipid components of EMCV ROs

Using a pharmacological inhibitor of PI4KA, we prove that EMCV and SAFV, which belong to distinct cardiovirus species, both require the lipid kinase activity for replication. In agreement with this result, we observed elevated PI4P levels at intracellular membranes in infected cells, suggesting an important role of PI4P lipids in cardiovirus replication. In non-infected cells, OSBP plays a critical role in lipid homeostasis by exchanging cholesterol for PI4P at the interface of ER and Golgi membranes, to which it localizes under normal conditions [20]. In this process, PI4P lipids also serve as a membrane anchor for OSBP. We hypothesized that in cardiovirus infection, PI4P may serve to recruit OSBP and cholesterol to viral replication sites. Indeed, OSBP was present at EMCV ROs, where it colocalized with the viral protein 3AB. This colocalization was markedly reduced upon AL-9 treatment, demonstrating that OSBP is recruited through PI4KA-produced PI4P. OSBP is an essential cardiovirus host factor, since both genetic depletion by siRNA treatment and pharmacological inhibition by OSW-1 and 25-HC blocked viral genome replication. Cholesterol was redistributed to EMCV ROs upon infection, and treatment with AL-9 or OSW-1 resulted in a significantly reduced colocalization of cholesterol with 3AB, arguing that accumulation of cholesterol at ROs is mediated by both PI4KA and OSBP. These data are in agreement with our recent findings that cholesterol shuttling is important for cardiovirus genome replication [54] and that cardioviruses are sensitive to itraconazole, which we recently discovered to be an. inhibitor of OSBP [65].

Our results indicate that PI4P and cholesterol are vital for the global organization of EMCV ROs. However, as these lipids fulfill multiple functions in various cellular processes [53,69–71], other roles in virus replication should be envisaged. A potential task of PI4P in virus replication may be linked to the PI(4,5)P_2_ synthesis pathway, since PI4P is the major precursor of PI(4,5)P_2_ lipids, which were recently attributed an important role in HCV replication [72]. Cholesterol homeostasis was recently shown to play an important role in efficient PV polyprotein processing [73]. Whether cholesterol also ensures a proper microenvironment that supports cardiovirus polyprotein processing remains to be determined. Interestingly and in apparent parallel with the distantly related enteroviruses, exploitation of the PI4K-OSBP pathway by HCV correlates with the induction of membranes of positive curvature [58]. By contrast, the flavivirus DENV, although closely related to HCV, does not require PI4K or OSBP [23] and generates membranes of negative curvature [74]. Hence, the interplay between PI4P and cholesterol may dictate the positive curvature of the membranes at which diverse RNA viruses replicate their genomes.

Through co-IP assays, we identified PI4KA as a novel interaction partner of EMCV proteins 3A and its precursor 3AB. EMCV 3A is a small protein (88 amino acids) of unknown structure, containing a predicted hydrophobic domain in the C-terminus half. Expression of 3A alone was sufficient for PI4KA recruitment in intact cells, arguing that in infection, PI4KA is recruited to ROs by this viral protein. Enteroviruses and kobuviruses recruit PI4KB to ROs also via their 3A protein [15,16,45,57], raising the possibility that diverse picornaviruses might use an evolutionary conserved and 3A-mediated mechanism to generate PI4P-enriched membranes. However, the 3A proteins of entero-, kobu- and cardioviruses do not share any apparent sequence similarity, their name simply reflecting the occupancy of the same locus (3A) in the respective viral genomes. With the exception of their catalytic domain, also the PI4KA and PI4KB isoforms do not share any sequence similarity [75]. Furthermore, unlike 3A of most enteroviruses, cardiovirus 3A does not interact with GBF1 nor blocks protein transport in the secretory pathway when expressed alone [76], highlighting the functional diversification associated with these small viral proteins.

### EMCV and HCV converged to recruit a common lipid-modifying pathway in building replication sites

Several lines of evidence suggest that the picornavirus EMCV and the distantly related flavivirus HCV have evolved to exploit common host components in assisting virus RNA replication. First, HCV genome replication occurs at the “membranous web” (MW), a network of single and DMVs that, like the EMCV RO, also mainly originates from the ER [58]. Second, both EMCV and HCV express a viral protein dedicated to recruitment of PI4KA, in order to induce a PI4P-rich environment at the replication sites [24,46]. Third, in HCV infection, PI4P lipids were also shown to be important for the recruitment of OSBP, which mediates cholesterol transfer to the MW [23]. Fourth, inhibition of either PI4KA or OSBP induced clear alterations in the global distribution of EMCV ROs, which appeared more “clustered” upon treatment with AL-9 or OSW-1. A similar clustering effect was also observed for replication structures of the HCV MW upon PI4KA or OSBP inhibition [23,24], whereas no obvious disruption of the enterovirus ROs was observed upon PI4KB or OSBP inhibition [18,19,65]. Together, these observations indicate that EMCV and HCV replication structures share critical host components, and possibly also a similar architecture, although the latter still remains to be determined.

**Fig 8:**
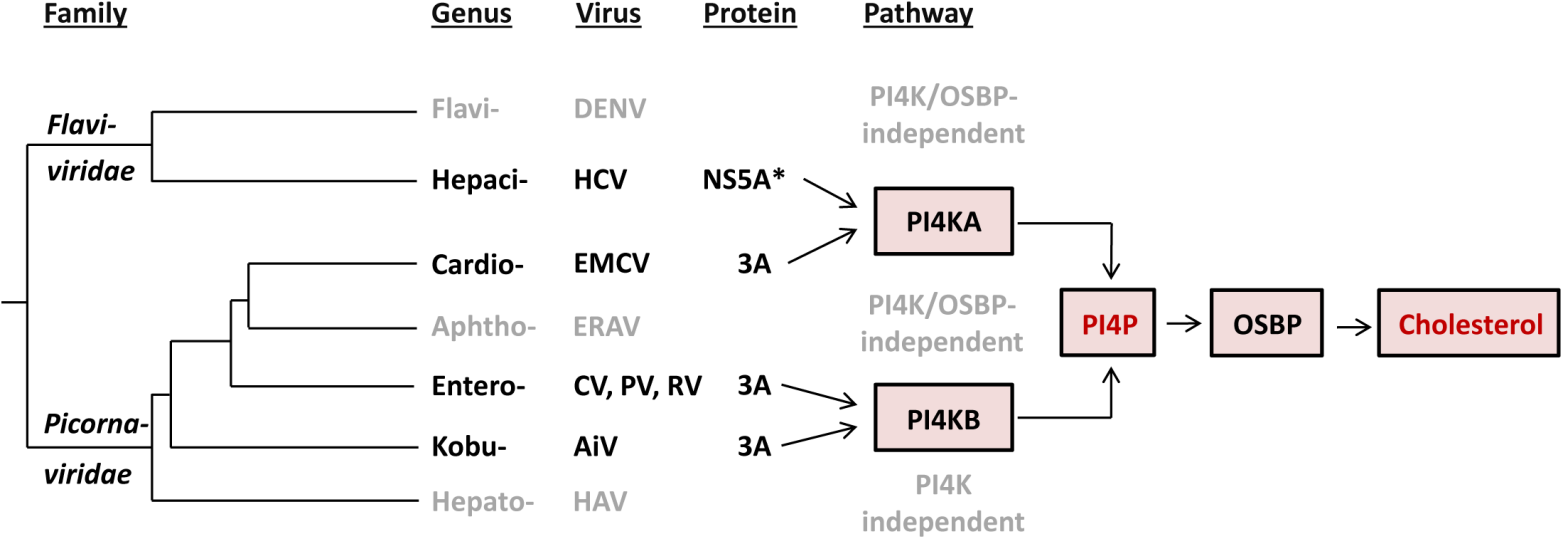
Evolution of the recruitment of lipid-modulating pathways by picorna- and flaviviruses. Our current understanding of the major elements of lipid-modulating pathways targeted by proteins of picorna- and flaviviruses in the context of the virus phylogeny is presented. Only viruses characterized so far with respect to this function are depicted. The tree branches are of arbitrary scale, while the branching of picornaviruses reflects the topology of maximum-likelihood RdRp-based tree according to Gorbalenya & Lauber, 2010 [80], and the entire tree is rooted according to Gibrat et al., 2013 [81]. *In addition to NS5A, NS5B has also been suggested to be involved in the interaction with PI4KA [24,46]. Abbreviation of virus names are as follows: DENV, dengue virus; HCV, hepatitis C virus; EMCV, encephalomyocarditis virus; ERAV, equine rhinitis A virus; CV, coxsackievirus; PV, poliovirus; RV, rhinovirus; AiV, Aichivirus; HAV, hepatitis A virus.

Based on at least two lines of emerging evidence in the context of phylogeny of flavi- and picornaviruses, EMCV and HCV have likely converged on rather than retained their functional similarities upon divergence from the common ancestor (Fig 8). First, the observed commonalities between EMCV and HCV are not shared by other characterized viruses in their respective families, indicating that they are not a manifestation of the properties conserved among the two families. For instance, picornaviruses from different genera exhibit different host factor requirements. Members of the *Cardiovirus* genus hijack the ER-localized PI4KA (this study), whereas members of the *Enterovirus* and *Kobuvirus* genera depend on the Golgi-localized PI4KB [15,34,57]. In contrast, equine rhinitis A virus (ERAV, member of *Aphthovirus* genus, which is prototyped by FMDV) and hepatitis A virus (*Hepatovirus* genus) seem to replicate independent of both PI4KB and PI4KA (S7 Fig and [34,77]). Likewise, the flaviviruses DENV and WNV, representing a sister genus to that of HCV, do not rely on either PI4KA or PI4KB [23,78]. While DENV was also shown not to require OSBP [23], for WNV this is not known yet. Importantly, cardioviruses targeting PI4KA occupy a lineage that is farther from the root compared to those of entero- and kobuviruses targeting PI4KB (Fig 8). This phylogenetic pattern is indicative of the relatively recent emergence of the EMCV-specific target properties. Second, EMCV and HCV employ apparently unrelated proteins to mediate the interaction with PI4KA, namely 3A and NS5A (although HCV NS5B may contribute as well [24,46]). Both proteins are membrane-bound, albeit through a hydrophobic domain located at either N-terminus (NS5A) or in the C-terminus-half (3A), and each includes another region which is among the least conserved in the nonstructural proteins of the respective families [79,80].

Our study contributes to the hypothesis that viruses may be confronted with powerful constraints that limit the diversity of host pathways recruited for efficient replication. Thus, a common pathway is used by different RNA viruses that either only moderately diverged (e.g. different species of same genus) or converged on a host target while diverging profoundly (different families–e.g. EMCV and HCV). To date, only a small number of (+)RNA viruses have been studied in terms of host lipid requirements. Identification of the lipid pathways used by other viruses will hopefully provide a deeper insight on the constraints that viruses are confronted with during the endeavor to replicate their genome.

## Materials and Methods

### Cells and reagents

Buffalo green monkey (BGM) kidney cells, baby hamster kidney 21 (BHK-21) and HeLa R19 cells were grown at 37°C and 5% CO_2_ in Dulbecco’s modified Eagle’s medium (DMEM, Lonza) supplemented with 10% fetal bovine serum (FBS). Huh7Lunet/T7 cells (provided by R. Bartenschlager, Department of Molecular Virology, University of Heidelberg, Heidelberg, Germany) [82] were grown in DMEM (Lonza) supplemented with 10% FBS and 10 μg/ml Blasticidin (PAA)_._ BGM cells were purchased from ECACC and BHK-21 cells were purchased from ATCC. HeLa R19 cells were obtained from G. Belov (University of Maryland and Virginia-Maryland Regional College of Veterinary Medicine, US) [83]. AL-9 and OSW-1 were kind gifts from J. Neyts (Rega Institute for Medical Research, University of Leuven, Leuven, Belgium) and M.D. Shair (Department of Chemistry and Chemical Biology, Harvard University, Cambridge, USA) respectively. 25-HC was purchased from Santa Cruz. BF738735 [84] was provided by Galapagos NV. Filipin III and dipyridamole were from Sigma.

### Plasmids

Constructs pTM-HA-PI4KA [24], pEGFP-PI4KA (provided by G. Hammond, NICHD, National Institutes of Health, Bethesda, USA) [41,85] and p3A(CVB3)-myc [86] were described previously. pGFP-PI4KB was a kind gift from N. Altan-Bonnet (Laboratory of Host-Pathogen Dynamics, National Institutes of Health, Bethesda, USA). To generate C-terminal myc-tagged EMCV proteins, genes encoding EMCV nonstructural proteins 2A, 2B, 2C, 3A, 3AB, 3C and 3D were amplified by PCR using the plasmid pM16.1 [87] and primers introducing restriction sites BamHI and HindIII. pM16.1 contains the full-length infectious cDNA sequence of EMCV, strain mengovirus. The PCR products were then cloned into the p3A(CVB3)-myc backbone from which the CVB3-3A gene was removed using the same restriction enzymes. To allow ectopic expression of PI4KA under a CMV promoter, the gene encoding HA-PI4KA was amplified by PCR using pTM-HA-PI4KA as template and introduced in the pEGFP-N3 backbone using restriction enzymes SalI and NotI. EMCV-2C-HA infectious clone was generated by introducing the HA coding sequence (YPYDVPDYA) in-frame after codon 2 in 2C of pM16.1 using mutagenesis primers and the Q5 Site-Directed Mutagenesis Kit (New England Biolabs).

### Viruses and infections

EMCV, EMCV-2C-HA and RLuc-EMCV, which contains the *Renilla* luciferase gene upstream of the capsid coding region [54], were obtained by transfecting BHK-21 cells with RNA transcripts derived from full length infectious clones pM16.1, pM16.1-2C-HA and pRLuc-QG-M16.1, respectively, linearized with BamHI. GFP-EMCV, which contains the EGFP gene upstream the capsid region, was generated similar as RLucEMCV [54]. CVB3 (strain Nancy) was obtained by transfecting BGM cells with RNA transcripts of the full length infectious clone p53CB3/T7 [86] linearized with SalI. Saffold virus (type 3) was described previously [2]. ERAV (NM11/67) was kindly provided by David Rowlands and Toby Tuthill (University of Leeds, United Kingdom). Virus infections were performed by incubating subconfluent cell monolayers for 30 min at 37°C with virus, after which the virus-containing medium was removed and fresh (compound-containing) medium was added to the cells (t = 0). In the time-of-addition experiments, medium without compound was added at t = 0 and replaced by medium with compound at the indicated time points. At the given time points post infection, cells were either fixed for immunolabeling, freeze-thawed to determine virus titers or, in the case of RLuc-EMCV, lysed to determine replication by measuring the intracellular *Renilla* luciferase activity using the *Renilla* Luciferase Assay System (Promega). Virus titers were determined by endpoint titration according to the method of Reed and Muench and expressed as 50% tissue culture infective doses (TCID50).

### Immunofluorescence microscopy

HeLa R19 or Huh7Lunet/T7 cells were grown to subconfluency on coverslips in 24-well plates. Where indicated, cells were transfected with 400 ng of plasmids using Lipofectamine2000 according to the manufacturer’s protocol and/or infected with EMCV at the specified multiplicity of infection (MOI), followed by compound treatment where specified. At the indicated time points, cells were fixed with 4% paraformaldehyde (PFA) for 20 min at room temperature (RT). Permeabilization was done with PBS-0.5% Triton X-100 for 15 min or PBS/0.2% saponin/5% BSA for 5 min, in the case of filipin staining. Cells were incubated sequentially with primary and secondary antibodies diluted in PBS containing 2% normal goat serum (NGS). The following primary antibodies were used for detection: mouse monoclonal anti-GM130 (BD Biosciences), rabbit polyclonal anti-TGN46 (Novus Biologicals), mouse monoclonal anti-ERGIC53 (Enzo Life Sciences), rabbit polyclonal anti-Sec13 (kindly provided by B.L Tang, Department of Biochemistry, The National University of Singapore, Singapore), rabbit polyclonal anti-calreticulin (Sigma), rabbit polyclonal anti-HA (Santa Cruz), mouse monoclonal anti-HA (Abcam), mouse monoclonal anti-C-Myc (Sigma), rabbit polyclonal anti-myc (Thermo Scientific), mouse anti-PI4P IgM (Echelon Biosciences), mouse monoclonal anti-dsRNA (J2, English & Scientific Consulting), mouse monoclonal anti-EMCV 3AB (kind gift from A.G. Aminev) [88] and rabbit polyclonal anti-OSBP (kindly provided by M.A. De Matteis, Telethon Institute of Genetics and Medicine, Naples, Italy) [65]. Alexa Fluor 488-, 594-conjugated IgG and Alexa Fluor 488- or 594-conjugated IgM (Invitrogen, Molecular Probes) were used as secondary antibodies. Cholesterol was stained with 25 μg/ml filipin III (Sigma) for 1 h at room temperature, included during the incubation with the secondary antibody. Nuclei were counterstained with DAPI.

Staining of plasma membrane or intracellular PI4P was performed as described elsewhere [50]. Briefly, for PM staining, cells were fixed at RT in 4% PFA and 0.2% glutaraldehyde. All subsequent steps were performed on ice. Cells were blocked and permeabilized for 45 min in buffer A (20mM Pipes, pH 6.8, 137 mM NaCl, 2.7 mM KCl) containing 5% NGS, 50 mM NH_4_Cl and 0.5% saponin. Slides were incubated with primary and secondary antibodies in buffer A containing 5% NGS and 0.1% saponin for 1 h. Finally, slides were post-fixed in 2% PFA in PBS for 10 min. The intracellular PI4P staining was performed at RT as follows: cells were fixed with 2% PFA, then permeabilized for 5 min in 20 μM digitonin in buffer A, blocked for 45 min in buffer A with 5% NGS and 50 mM NH_4_Cl and then incubated sequentially with primary and secondary antibodies in buffer A with 5% NGS, before post fixation in 2% PFA. All coverslips were mounted with FluorSave (Calbiochem). Images were acquired with a Leica SPE-II DMI-4000 confocal laser scanning microscope or a Nikon Ti Eclipse microscope equipped with an Andor DU-897 EMCCD-camera.

### Image analysis

PI4P quantification was performed for at least 40 cells for each condition, using the ImageJ software as described elsewhere [46]. To determine colocalization of Sec13 or calreticulin with 3AB, images were first deconvoluted using NIS advanced Research 4.3 software (Nikon) (10 iterations) and further processed using Image J as follows. Individual infected cells were outlined and a mask was created, and all signal outside the mask was cropped to exclude it from the calculations. Manders’ colocalization coefficient was calculated for at least 10 cells for each condition using the JACoP plugin [89] with a manually set threshold. Colocalization of OSBP with 3A in infected cells was analyzed using ImageJ by determining Pearson’s coefficient for at least 15 cells per condition using the Coloc 2 plugin with default settings. To quantify colocalization of filipin with 3AB, images were first deconvoluted using NIS software (20 iterations), then ImageJ was used to select infected cells and the Pearson’s coefficient of colocalization for at least 15 cells per condition was calculated using the Coloc 2 plugin with default settings.

### siRNA treatment

HeLa R19 cells were reverse-transfected with 2 pmoles of siRNA per well of a 96-well plate (2000 cells/well) using Lipofectamine 2000 (Invitrogen) according to the manufacturer’s indications. Scrambled siRNA (AllStars Neg. Control, Qiagen) was used as a control. SiRNA against hPI4KA (cat. no. S102777390) and hPI4KB (target sequence: 5’-UGUUGGGGCUUCCCUGCCCTT-3’) were from Qiagen. siRNA against hOSBP (two siRNAs mixed at 1:1 ratio, target sequences: 5’- CGCUAAUGGAAGAAGUUUA[dT][dT]-3’ and 5’-CCUUUGAGCUGGACCGAUU[dT][dT]-3’)) was from Sigma. 48 h p.t., cells were either infected with virus, transfected with *in vitro* transcribed RNA derived from the full length infectious clone pM16.1 or harvested to evaluate the knockdown efficiency by western blot analysis.

### Cell viability assay

Cell viability was determined in parallel with virus infection as follows. One day after seeding cells in a 96-well plate, the compounds were added to the cells and incubated for 8 h. Alternatively, cells were transfected with siRNAs and incubated for 48 h. Subsequently, the medium was replaced with CellTiter 96 AQueous One Solution Reagent (Promega) and optical densities were measured at 490 nm. The obtained raw values were converted to percentage of untreated samples or samples transfected with scrambled siRNAs, following correction for background absorbance.

### Radioactive labeling and immunoprecipitation

Metabolic labeling of myc-tagged EMCV proteins and HA-PI4KA was performed as described elsewhere [46]. Briefly, Huh7-Lunet/T7 cells seeded in 6-well plates were co-transfected with 2 μg of plasmid encoding EMCV nonstructural proteins and 2 μg of either pTM HA-PI4KIIIa or an empty pTM vector (mock) using Lipofectamine2000 (Invitrogen) according to the manufacturer’s instructions. 7 h later, cells were starved in methionine/cysteine-free medium for 1 h. Radiolabeling of cells was done by overnight incubation in methionine/cysteine-free medium, supplemented with 10 mM glutamine, 10 mM Hepes, and 100 μCi/ml of Express Protein labeling mix (Perkin Elmer, Boston). Cells were then harvested and lysed in lysis buffer (50 mM Tris-Cl [pH 7.5], 150 mM NaCl, 1% Nonidet P-40 and protease inhibitors) for 1 h on ice, followed by centrifugation at 14,000 g for 10 min at 4°C. Supernatants were further subjected to immunoprecipitation by a 3 h incubation at 4°C with anti-c-myc rabbit polyclonal antibody (Santa Cruz). Immunocomplexes were then captured with protein G-sepharose beads (Sigma) by an additional 3 h incubation at 4°C. Beads were washed three times in lysis buffer, followed by elution of immunocomplexes by boiling in sample buffer, separation by polyacrylamide-SDS gel electrophoresis and detection by autoradiography. For co-IP followed by western blot, cells were seeded in 55 cm^2^ dishes and transfected with 3.5 μg of each plasmid using polyethylenimine (PEI) (Polysciences). Immunoprecipitation was carried out as described above, but using protein A-sepharose beads (GE Healthcare) and mouse monoclonal anti-C-Myc (Sigma) or rabbit polyclonal anti-myc (Thermo Scientific) antibodies.

### Western blot analysis

Samples separated by SDS-PAGE were transferred to nitrocellulose membranes (Bio-Rad). Membranes were incubated with the following primary antibodies: rabbit polyclonal anti-PI4KA (Cell Signaling), rabbit polyclonal anti-PI4KB (Upstate), rabbit polyclonal anti-OSBP (ProteinTech), rabbit polyclonal anti-EMCV capsid (kind gift from Ann Palmenberg) and mouse monoclonal anti-β-actin (Sigma). Secondary antibodies included IRDye 680-conjugated goat anti-mouse or IRDye 800-conjugated goat anti-rabbit (LI-COR). Images of blots were acquired with an Odyssey Fc Imaging System (LI-COR).

### Statistical analyses

Where indicated, unpaired one-tailed Student’s t-test or two-tailed Mann–Whitney test were applied as statistical analyses using the GraphPad Prism software.

## Supporting Information

S1 Fig AL-9 inhibits EMCV at the step of genome replication. (A) AL-9 is effective against EMCV also at high MOI. HeLa R19 cells were infected with RLuc-EMCV at an MOI of 10 followed by AL-9 treatment for 8 h, after which cells were lysed and virus replication was measured by determining the *Renilla* luciferase activity. (B) AL-9 blocks EMCV genome replication. HeLa R19 cells were infected with RLuc-EMCV at an MOI of 1. At the indicated time points, DMSO, 10 μM AL-9 or 100 μM dipyridamole was added to the cells and virus replication was measured by determining the *Renilla* luciferase activity at 8 h p.i. Bars represent mean values of triplicates ± standard error of the means (SEM). Means were statistically compared using unpaired *t* tests. *P < 0.05, **P<0.01; ***P<0.001.
(TIF)

S2 Fig EMCV 3A recruits PI4KA to replication sites. HeLa R19 cells were transfected with a plasmid encoding HA-PI4KA and infected the following day with EMCV at MOI 250. At 6 h p.i., cells were fixed and co-stained with antibodies against HA and viral 3AB. Nuclei were stained with DAPI (blue). The crop panels at the bottom depict enlargements of boxed areas. Scale bars represent 10 μm.
(TIF)

S3 Fig Characterization of EMCV-2C-HA. (A) Schematic representation of EMCV-2C-HA genome organization drawn to scale (nucleotide base length). The HA tag (YPYDVPDYA) was introduced in frame after the second amino acid in the viral protein 2C. (B) Replication kinetics of EMCV-2C-HA compared to wt EMCV. HeLa R19 cells were infected at MOI 1 with EMCV or EMCV-2C-HA. At the indicated time points p.i., cells were freeze-thawed to release intracellular virus particles and the total virus titers were determined by endpoint titration. Shown are mean values ± SEM. (C) 2C-HA is present at 3AB-positive replication membranes. Huh7-Lunet/T7 cells were infected with EMCV-2C-HA at MOI 10. At 6 h p.i., cells were fixed and co-stained with antibodies against HA and viral 3AB. Nuclei were stained with DAPI (blue). The crop panels at the bottom depict enlargements of boxed areas. Scale bars represent 10 μm.
(TIF)

S4 Fig Time course analysis of endogenous PI4KA in EMCV-infected cells. Huh7-Lunet/T7 cells or HeLa R19 cells were infected with EMCV at MOI 10. At the indicated time points p.i., cells were lysed and the whole-cell lysates were subjected SDS-PAGE followed by western blot analysis using specific antibodies against actin, EMCV capsid or endogenous PI4KA. For PI4KA visualization, samples were separated in a resolving SDS-PAGE gel composed of two different acrylamide concentrations: 7.5% (for the upper half) and 15% (for the lower half).
(TIF)

S5 Fig EMCV alters the distribution of intracellular PI4P lipids. HeLa R19 cells were mock-infected or infected with GFP-EMCV at MOI 10. At 6 h p.i., cells were fixed and stained with antibodies against PI4P using specific protocols for detection of plasma membrane and intracellular PI4P pools [50]. Nuclei were stained with DAPI (blue). Scale bars represent 10 μm.
(TIF)

S6 Fig OSW-1 inhibits EMCV replication also at high MOI. HeLa R19 cells were infected with RLuc-EMCV at an MOI of 10 followed by OSW-1 treatment for 8 h, after which cells were lysed and virus replication was measured by determining the *Renilla* luciferase activity. Bars represent mean values of triplicates ± standard error of the means (SEM). Means were statistically compared using unpaired *t* tests. *P < 0.05, **P<0.01; ***P<0.001.
(TIF)

S7 Fig Aphthoviruses are insensitive to AL-9 treatment. HeLa R19 cells were infected with ERAV or EMCV at MOI 1 followed by treatment with AL-9 at the indicated concentrations. Cells were freeze-thawed to release intracellular virus particles and the total virus titers at 8 h p.i. (EMCV) or 24 h p.i. (ERAV) were determined by endpoint titration. For ERAV, the highest concentration of AL-9 used was 5 μM in order to avoid cytotoxicity. Dashed lines represent input levels. Shown are mean values ± SEM.
(TIF)
