# Supplementary figures and images for: Modulation of the Host Lipid Landscape to Promote RNA Virus Replication: The Picornavirus Encephalomyocarditis Virus Converges on the Pathway Used by Hepatitis C Virus
Source: PLoS Pathog. 2015 Sep 25;11(9):e1005185. doi: 10.1371/journal.ppat.1005185 (PMC4583462; doi:10.1371/journal.ppat.1005185)

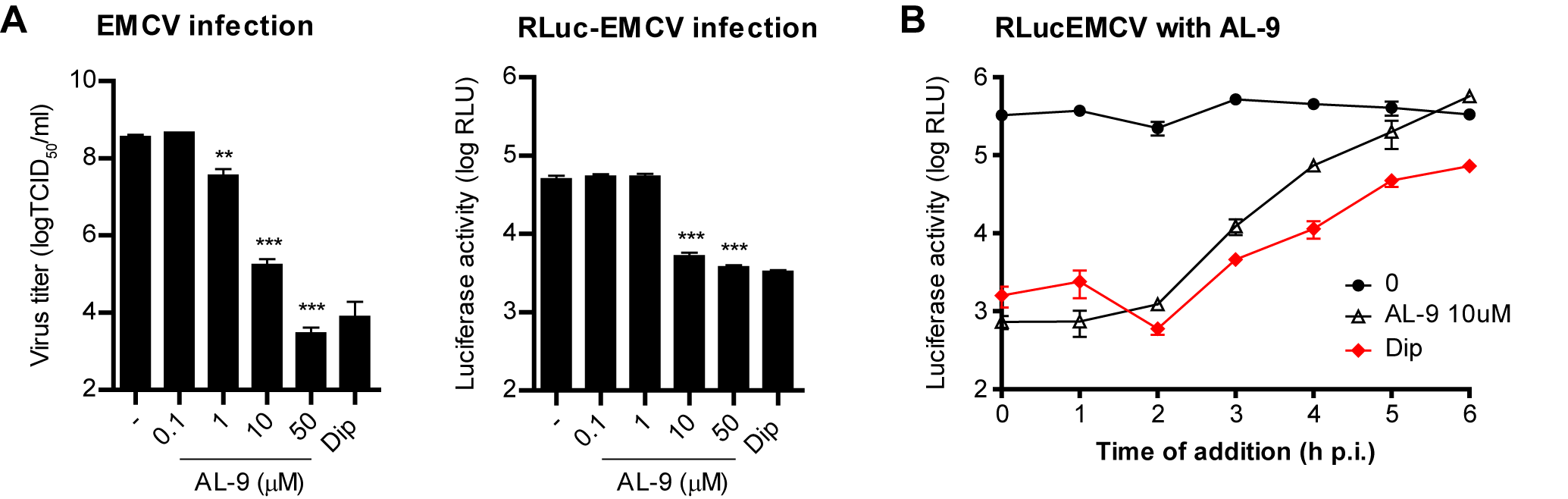

Supplement: S1 Fig — (A) AL-9 is effective against EMCV also at high MOI. HeLa R19 cells were infected with RLuc-EMCV at an MOI of 10 followed by AL-9 treatment for 8 h, after which cells were lysed and virus replication was measured by determining the Renilla luciferase activity. (B) AL-9 blocks EMCV genome replication. HeLa R19 cells were infected with RLuc-EMCV at an MOI of 1. At the indicated time points, DMSO, 10 μM AL-9 or 100 μM dipyridamole was added to the cells and virus replication was measured by determining the Renilla luciferase activity at 8 h p.i. Bars represent mean values of triplicates ± standard error of the means (SEM). Means were statistically compared using unpaired t tests. *P < 0.05, **P<0.01; ***P<0.001. (TIF) [file ppat.1005185.s001.tif]

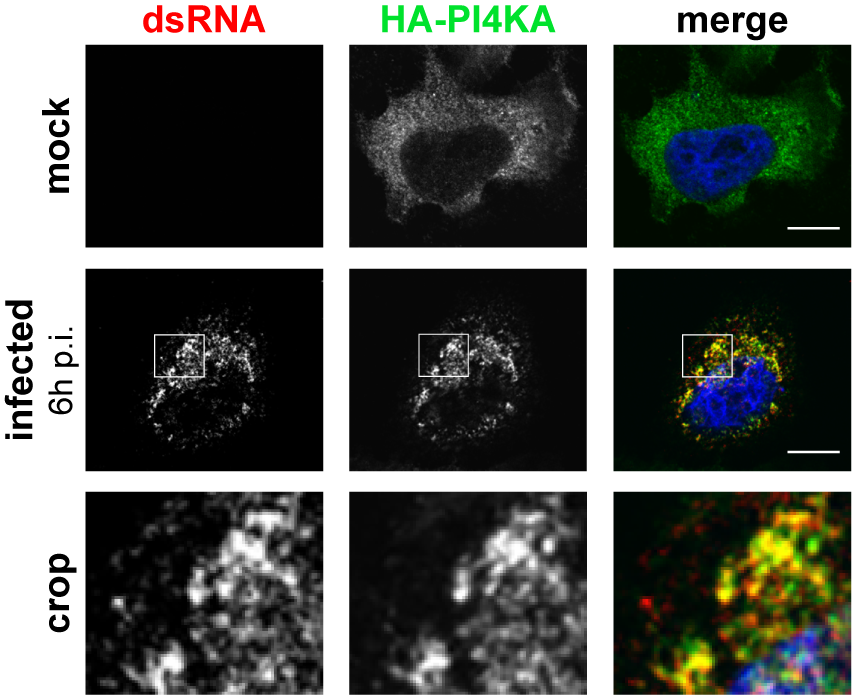

Supplement: S2 Fig — HeLa R19 cells were transfected with a plasmid encoding HA-PI4KA and infected the following day with EMCV at MOI 250. At 6 h p.i., cells were fixed and co-stained with antibodies against HA and viral 3AB. Nuclei were stained with DAPI (blue). The crop panels at the bottom depict enlargements of boxed areas. Scale bars represent 10 μm. (TIF) [file ppat.1005185.s002.tif]

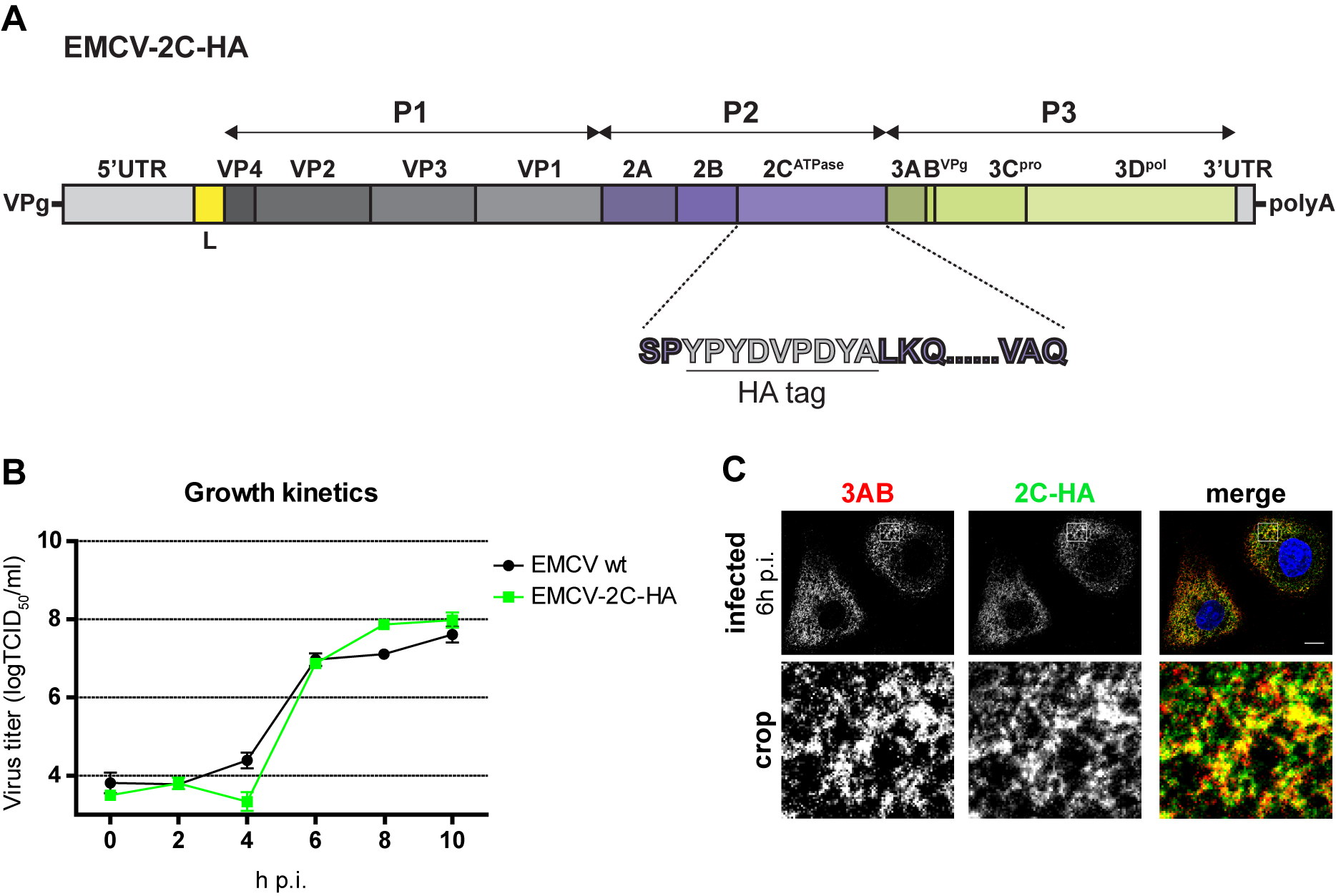

Supplement: S3 Fig — (A) Schematic representation of EMCV-2C-HA genome organization drawn to scale (nucleotide base length). The HA tag (YPYDVPDYA) was introduced in frame after the second amino acid in the viral protein 2C. (B) Replication kinetics of EMCV-2C-HA compared to wt EMCV. HeLa R19 cells were infected at MOI 1 with EMCV or EMCV-2C-HA. At the indicated time points p.i., cells were freeze-thawed to release intracellular virus particles and the total virus titers were determined by endpoint titration. Shown are mean values ± SEM. (C) 2C-HA is present at 3AB-positive replication membranes. Huh7-Lunet/T7 cells were infected with EMCV-2C-HA at MOI 10. At 6 h p.i., cells were fixed and co-stained with antibodies against HA and viral 3AB. Nuclei were stained with DAPI (blue). The crop panels at the bottom depict enlargements of boxed areas. Scale bars represent 10 μm. (TIF) [file ppat.1005185.s003.tif]

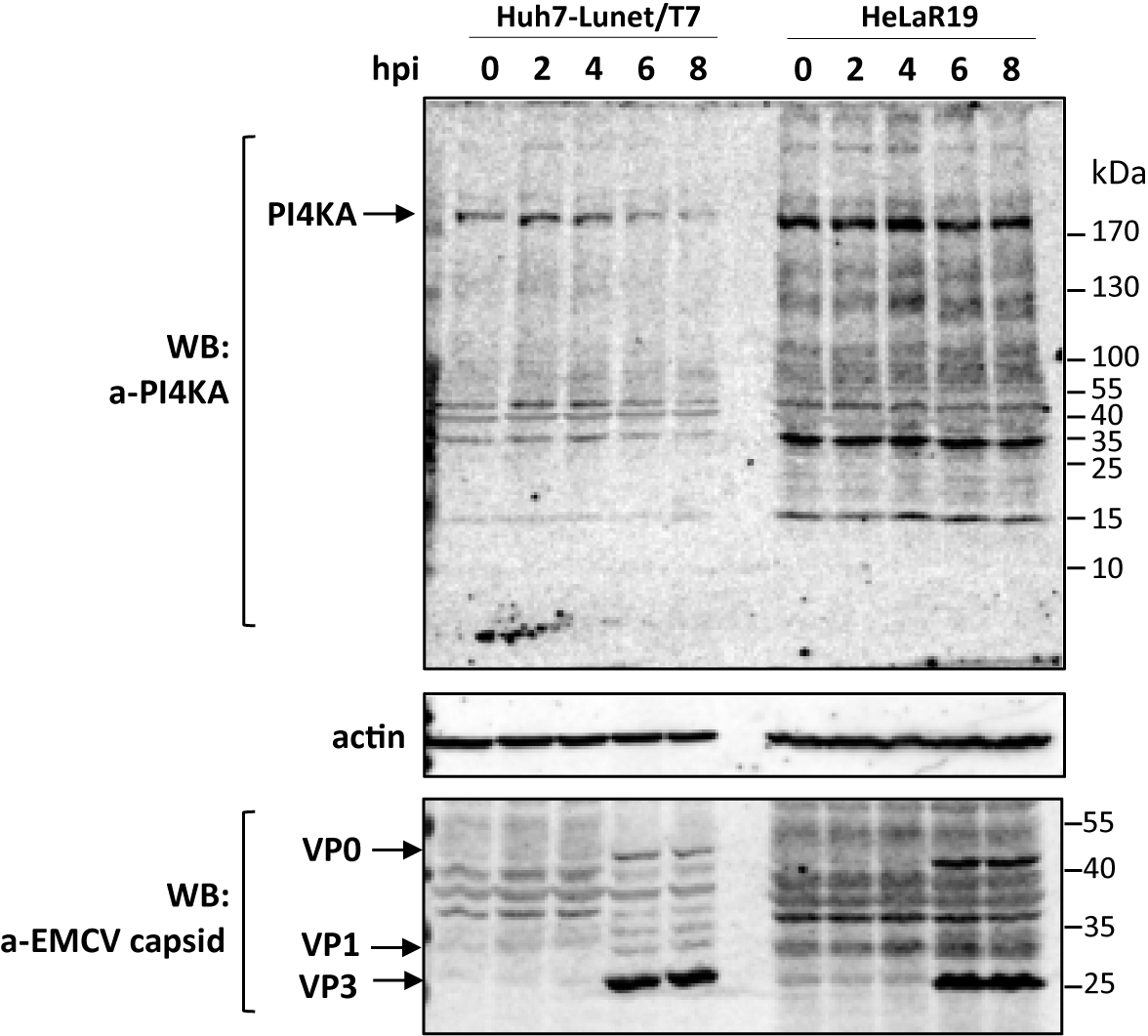

Supplement: S4 Fig — Huh7-Lunet/T7 cells or HeLa R19 cells were infected with EMCV at MOI 10. At the indicated time points p.i., cells were lysed and the whole-cell lysates were subjected SDS-PAGE followed by western blot analysis using specific antibodies against actin, EMCV capsid or endogenous PI4KA. For PI4KA visualization, samples were separated in a resolving SDS-PAGE gel composed of two different acrylamide concentrations: 7.5% (for the upper half) and 15% (for the lower half). (TIF) [file ppat.1005185.s004.tif]

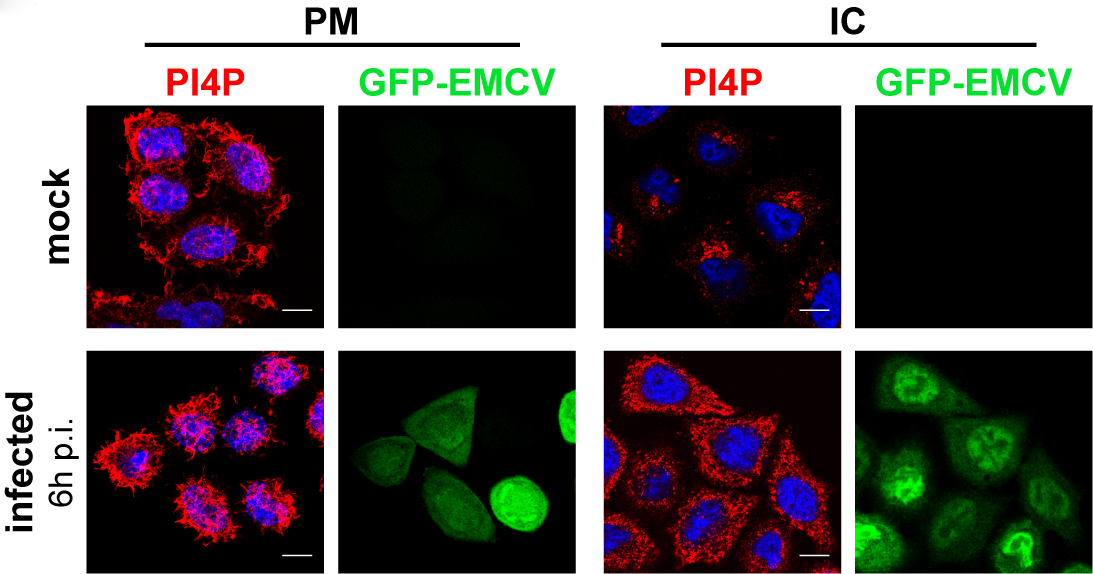

Supplement: S5 Fig — HeLa R19 cells were mock-infected or infected with GFP-EMCV at MOI 10. At 6 h p.i., cells were fixed and stained with antibodies against PI4P using specific protocols for detection of plasma membrane and intracellular PI4P pools [50]. Nuclei were stained with DAPI (blue). Scale bars represent 10 μm. (TIF) [file ppat.1005185.s005.tif]

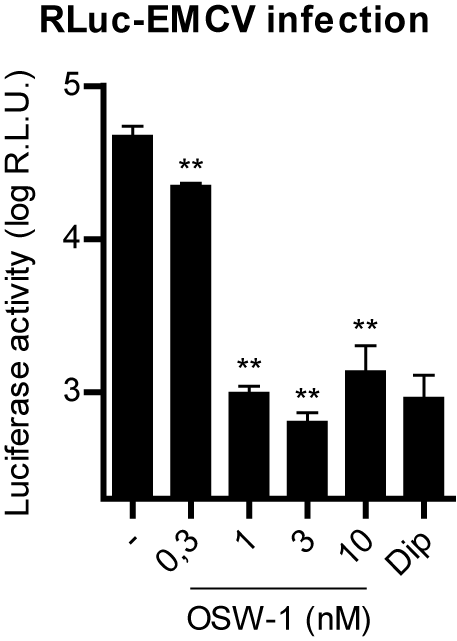

Supplement: S6 Fig — HeLa R19 cells were infected with RLuc-EMCV at an MOI of 10 followed by OSW-1 treatment for 8 h, after which cells were lysed and virus replication was measured by determining the Renilla luciferase activity. Bars represent mean values of triplicates ± standard error of the means (SEM). Means were statistically compared using unpaired t tests. *P < 0.05, **P<0.01; ***P<0.001. (TIF) [file ppat.1005185.s006.tif]

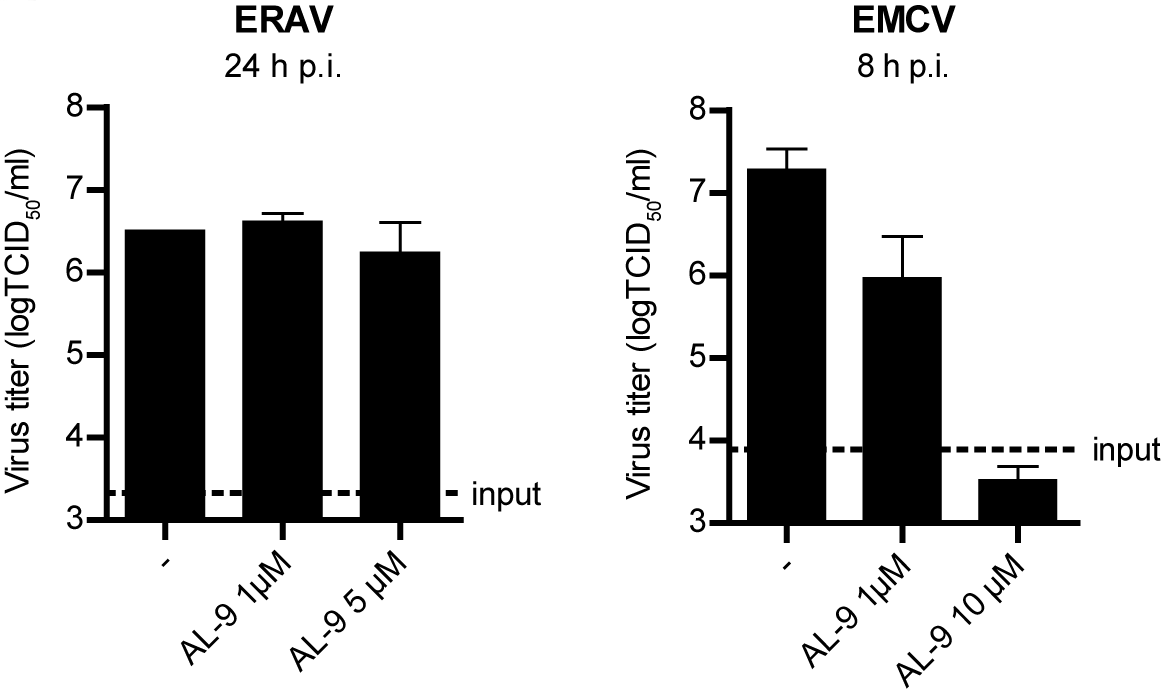

Supplement: S7 Fig — HeLa R19 cells were infected with ERAV or EMCV at MOI 1 followed by treatment with AL-9 at the indicated concentrations. Cells were freeze-thawed to release intracellular virus particles and the total virus titers at 8 h p.i. (EMCV) or 24 h p.i. (ERAV) were determined by endpoint titration. For ERAV, the highest concentration of AL-9 used was 5 μM in order to avoid cytotoxicity. Dashed lines represent input levels. Shown are mean values ± SEM. (TIF) [file ppat.1005185.s007.tif]
